# Osteoclast-like multinucleated giant cells reinforce polycaprolactone grafts

**DOI:** 10.3389/fimmu.2025.1572238

**Published:** 2025-05-21

**Authors:** Halldór Bjarki Einarsson, Anders Frisk Mortensen, Morten Schallburg Nielsen, Menglin Chen, Søren Roesgaard Nielsen, David Christian Evar Kraft, Jonas Jensen, Mette Bjerre, Morten Nørregaard Andersen, Jens Vinge Nygaard, Cody Eric Bünger, Thomas Vorup-Jensen

**Affiliations:** ^1^ Department of Neurosurgery, Aalborg University Hospital, Aalborg, Denmark; ^2^ Department of Neurosurgery, Aarhus University Hospital, Aarhus, Denmark; ^3^ Department of Orthopaedic Surgery, Aarhus University Hospital, Aarhus, Denmark; ^4^ Department of Biomedicine, Aarhus University, Aarhus, Denmark; ^5^ Department of Biological and Chemical Engineering, Aarhus University, Aarhus, Denmark; ^6^ Interdisciplinary Nanoscience Center (iNANO), Aarhus University, Aarhus, Denmark; ^7^ Department of Dentistry and Oral Health, Aarhus University, Aarhus, Denmark; ^8^ Department of Neuroradiology, Aarhus University Hospital, Aarhus, Denmark; ^9^ Department of Clinical Medicine, Aarhus University, Aarhus, Denmark

**Keywords:** polycaprolactone implants, monocyte, multinucleated giant cells, integrins, dynamic mechanical analysis

## Abstract

Successful application of advanced engineered materials in osteoplasty requires a biological understanding of the recipient reaction. The immune system acts like a double-edged sword by maintaining targeted tissue and rejecting grafts. Nevertheless, even for promising graft materials such as polycaprolactone, insights on contact with immune cells have been restricted due to lacking quantitative assays. Here, we show that polycaprolactone graft sites after cranioplasty are dominated by an immature type of multinucleated giant cells, probably derived from transmigrating peripheral monocytes. The cells interact with the polycaprolactone through extensive pseudopodia formation and localized polymer dissolution. Dynamic mechanical analysis revealed osteoclast-like cells, derived *in vitro* from primary human monocytes, reinforce polycaprolactone by depositing a CD18 integrin-rich attachment matrix. Our findings give a new perspective on immune cells’ beneficial and detrimental functions in graft lesions, guiding therapy with better graft designs.

## Introduction

Skull defects are often repaired by reconstructive cranial surgery or cranioplasty. Nevertheless, a successful outcome is limited by the complex environment with intricate bone structure and functional forces presenting several challenges for reconstruction (1). While available procedures permit efficient graft acceptance and biocompatibility, the frequent need for revision surgery significantly increases the patient’s discomfort and can be life-threatening (2). The most common complication associated with cranioplasty is graft resorption. Still, our knowledge remains lacking on these processes’ cellular and molecular basis (3).

Polycaprolactone (PCL) is a hydrophobic semi-crystalline linear aliphatic polyester. It has desirable rheological properties and a low melting point at ~60°C (4, 5). With the rise of modern tissue engineering, there has been increased interest in PCL due to its biodegradability and its high suitability for 3D-printing, injection molding, and electrospinning (1, 6–11). Although not widely translated to the clinic (1), PCL already holds promise from its use in drug delivery vehicles, surgical sutures, cartilage repair, neurite outgrowth, and neurosurgical cranioplasty procedures in humans (4, 12–15). Polymethylmethacrylate remains the most frequently used allogenic material for cranial reconstruction surgery (16). However, over the past several years, individualized treatment has gained focus, and there is currently no consensus on which foreign body material is the most suitable for each patient regarding cranioplasty. This situation derives from missing insights on long-term outcomes and allogenic material comparisons in both animal models and humans. PCL implantation in cranioplasty is rare but has drawn more attention within the last ten years after the technical case report by Schantz et al. (12). In a recent human study on seven patients who underwent 3D-printed PCL/beta-tricalcium phosphate implant-based cranioplasty, a promising clinical outcome was revealed after 6 months. The implant showed sufficient mechanical properties and satisfactory cosmetic results (17). Comparable customized 3D-printed cranioplasty PCL implants with heparan sulfate glycosaminoglycan in Sprague-Dawley rats enhanced the bone healing efficacy (18). Furthermore, a recent case study using porous PCL meshes to cover a decompressive craniectomy defect demonstrated restoration of the natural bony contour without any postoperative complications at 1-year follow-up. The patient had undergone serial revision operations with titanium mesh and multiple treatments due to wound infections (19). A summary of these findings suggested that 3D-printed PCL and PCL/beta-tricalcium phosphate implants, incorporated with autologous cells and cytokines, may even facilitate cranial bone regeneration (20). This proposition has been supported by patient-specific cranioplasty PCL implants inoculated with bone marrow aspirate (21). Further, composite grafts of 3D PCL fiber mats and oil-based calcium phosphate cement pastes indicate a promising patient-specific degradable implant in maxillofacial and cranial surgery (22). However, mainly because there is a difference between degradability and resorbability for some polymers, reduction of biocompatibility or non-complete resorption of such material implants can have catastrophic implications for the patients (23, 24). These reactions originate mainly through inflammatory responses. Macroscopic inflammation was found at the *in-situ* tissue-PCL junctions following a 3D-printed trachea transplant in an animal model (25). Available data on PCL-induced inflammation includes histological observations of an elevated presence of T lymphocytes and myeloid CD14^+^ cells associated with woven and electro-spun PCL in a sheep tendon injury model (7). Earlier studies in rats also found an accumulation of CD14^+^ macrophages and unspecified multinucleated giant cells (MNGC) interacting with the PCL, including phagolysosomal material degradation (26). Both macrophages and multinucleated osteoclasts (OC) share monocytic precursors (27–29), but a possible link has not been substantiated between PCL-degrading myeloid cells in the bone and monocytes of the peripheral immune system.

PCL is hydrolyzed in aqueous acetonitrile solutions only by adding hydrochloric acid (HCl) (30). However, OCs can translocate acidic hydrolases and H^+^ across their cell membrane, forming HCl in the microenvironment (31–33). The ring-opening polymerization of repeating C_6_H_10_O_2_ monomers loads PCL with polycaprolactone-polyurethane ester bonds (34, 35). Cells resembling OCs exhibit a vigorous esterase activity (36). These cellular characteristics would suggest OCs as major degraders of PCL, but insight into this question is missing. Equally intriguing, OCs are positive for tartrate-resistant acid phosphatase (TRAP), similar to foreign-body giant cells (FBGCs) with resorption capabilities activated by T lymphocytes. Nevertheless, it is unclear what mechanisms are responsible for the marked transmigration of leukocytes into contact with the PCL grafts, if T lymphocytes play a role in OC activation in reaction to implants, or if PCL resorption could be subject to such regulation. *In toto*, the consequences of the immune involvement in PCL implants are unclear, even compared to the simpler spontaneous hydrolysis.

Integrins are essential cell adhesion molecules. In most leukocytes and leukocyte-derived cells such as OCs, β_2_ (CD18) integrins are strongly expressed. They deliver mechanical strength to maintain adhesion in blood vessels during leukocyte diapedesis through the endothelium and during phagocytosis of particulate materials of both natural and engineered origin. The integrins α_M_β_2_ (also known as Mac-1, complement receptor 3, CD11b/CD18) and α_X_β_2_ (complement receptor 4, CD11c/CD18) recognize denatured proteins, including denatured albumin and fibrinogen (37, 38). Recently, it was shown that ligation-induced signaling in these receptors plays has a significant role in priming acidified phagolysosomes with a high content of esterases (39, 40). Protein denaturation often occurs in biofouling processes where spontaneous adsorption may form stable and dynamic layers on top of polymeric materials (41, 42). Further, surface-induced protein denaturation often accompanies amyloid formation (43), another proteineous material now established as a potent ligand for CD18 integrins (40). Polymeric surfaces may also activate the complement system, with covalent deposition of the complement fragment iC3b, a primary CD11b/CD18 and CD11c/CD18 ligand (44). Several protein surface deposition processes explain how leukocytes via integrin adhesion are central to implant-mediated inflammation (45). However, the functional consequence of integrin binding to the implant materials is unclear.

Here, we address the current challenges of understanding the inflammatory environment created by PCL implants. We employ clinically relevant manufacturing approaches to obtain extrusion die-plotted plates or electro-spun fibers. Cranioplasty in pigs provokes monocyte recruitment differentiating to MNGCs with concomitant transmigration of T lymphocytes into PCL-filled defects. *In vitro*-differentiated OC-like MNGCs cell can degrade and resorb these materials. However, our quantitative assays revealed that spontaneous hydrolysis is the primary route of PCL degradation. By contrast, the leukocyte works to reinforce the PCL mechanically, apparently in a process involving shedding the CD18 integrins. The responses are surprisingly fast and have a dynamic impact, adding a new perspective on the future use of this material in cranioplasty in the context of immunomodulatory intervention.

## Results

### MNGCs formation and peripheral leukocyte transmigration into PCL implantation sites

**Figure 1:**
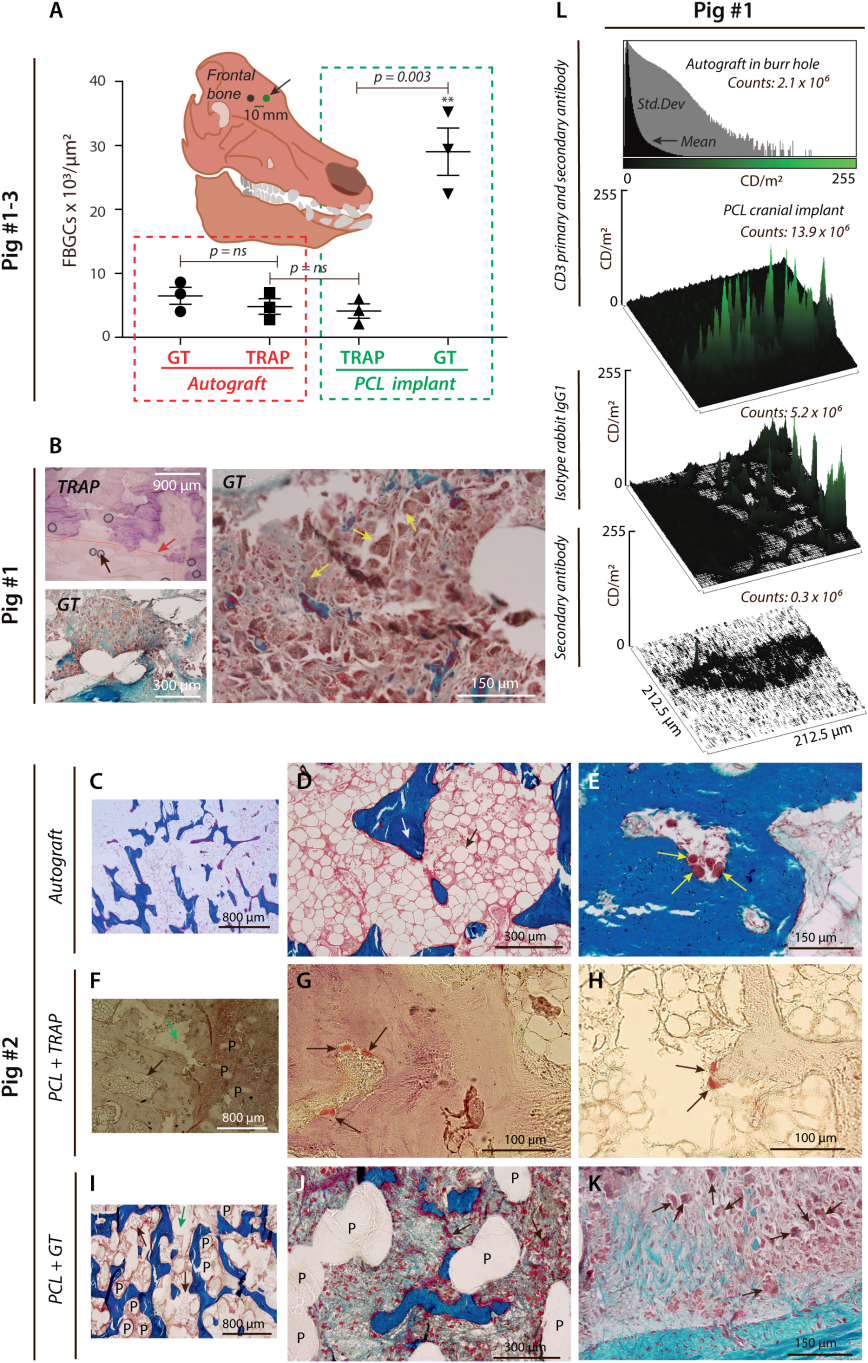
PCL provokes FBGC formation and T lymphocyte infiltration. **(A)**, Histological results were quantified from a paired porcine calvarial model. The number of FBGCs per µm^2^ is shown after biopsies taken from 3D-plotted PCL scaffold implants and extracted from the frontal cranial bone (black arrow). MNGCs were found to be predominantly TRAP- GT+ in the PCL group (p < 0.05, ANOVA), with a significant difference for TRAP^+^ cells vs. GT^+^ cells (green box) assessed in a two-sided *t*-test (n=3). **(B)**, Blinded gaiting strategy is shown (red line and red arrow). Scattered black circles on the TRAP-stained micro image (magnification ×4) are shown and represent artifacts (black arrow) caused by the sample preparation and the TRAP staining. The predominance of TRAP- GT+ cells observed at magnification ×4 and with severe bone marrow infiltration was histologically validated at magnification ×20 (yellow arrows). **(C–K)**, Induction of histopathology by PCL implants in cranioplasty. Representative GT-stained micro images at different magnifications **(C–E)**, showing normal trabecular bone (**(D)**, white arrow) and bone marrow (**(D)**, black arrow) after eight weeks from skull trepanation and implantation of the gained autograft placed into the burr hole (magnification ×4 and ×20). **(E)**, OC-like cells at the trabecular bone surface (yellow arrows). **(F–H)**, TRAP-stained specimen showing smooth PCL structures within the calvarial bone at Week 8 from PCL implantation (**(F)**, black arrow for bone marrow, green arrow showing an artefact, P for PCL fibers and at magnification ×10). At higher magnification (**(G)**, ×100), TRAP^+^ cells (black arrows) were detected on the bone surface. TRAP^+^ cells were also attached to a PCL debris-like structure **(H)** at magnification ×100 (black arrows). GT-stained specimen showing PCL fibers (*P*) between trabecular bone structures and bone marrow (black arrows) with a centrally placed artifact (green arrow) at magnification ×10 **(I)**. Imaging of smooth PCL fibers (*P*) within the same bone mass (magnification ×40), indicating an increased association of FBGCs (black arrows), which was further supported at higher magnification (×100, **(K)**). **(L)**, Semi-quantification by confocal microscopy of CD3 luminance signal shown by histograms and surface plots. Skull autograft in the cranial burr hole was compared with PCL, either stained with Ab to CD3, isotype control antibody, or secondary anti-IgG antibody.

Histological analyses on cranial PCL implants in pigs were stained with Goldner’s trichrome (GT) or TRAP stains to characterize the leukocyte populations responding to implantation. As a clinically relevant comparison, autografted bone was also included in parallel with PCL cranioplasty (**Figure 1A**).

From the histological assessment, it was evident that the number of GT+ MNCGs increased in the vicinity of the PCL implants (**Figure 1A**), and GT+ MNGCs in the PCL test groups also infiltrated the bone marrow (**Figure 1B**). TRAP+ MNGCs, suggesting a more mature osteoclast-like phenotype, were strikingly less abundant (**Figures 1A, B**), but clearly in contact with PCL (**Figures 1F–K**, **Supplementary Figures 1A–F**). By contrast, the control group receiving autograft had normal bone marrow and delineation of osteoclast-like cells on the surface of trabecular-like bone structures (**Figures 1C–E**).

The vast increase in GT+ MNGCs asked the question of the source of leukocytes. The phenotype of the MNGCs is close to other myeloid cells, including monocytes. Hence, it is difficult unambiguously to distinguish invading from resident myeloid cells. This observation is unlike the presence of lymphocytes, which are usually not present in healthy bone structures (46, 47). As a test for the presence of peripheral leukocytes in the PCL cranioplasty sites, we stained cells for CD3, the T lymphocyte receptor complex, expressed vigorously only in T lymphocytes and in some minor populations of natural killer cells. The staining confirmed the presence of T lymphocytes, suggesting that peripheral leukocytes entered the region with PCL (**Figure 1L**, **Supplementary Figure 1G**). A weak CD3 signal of 2.1×10^6^ counts/m^2^ was found for an autograft control. By contrast, a strong CD3 signal in two out of three donor pigs was found for the PCL implants with a signal of 13.9×10^6^ counts/m^2^. For comparison, signals of 5.2×10^6^ counts/m^2^ and 0.3×10^6^ counts/m^2^ were detected for PCL samples treated with isotype rabbit IgG1 antibody alone or, in addition, secondary antibody.

### *In vitro* generation of MNGCs with PCL reabsorption and phagocytotic capabilities

Based on the hypothesis from the cranioplasty analysis that peripheral monocytes are precursors of MNGCs *in vivo*, we wanted to understand the function of monocytes and differentiated MNGCs in PCL reabsorption and phagocytosis. Likewise, we wanted to include allotypic T lymphocytes to probe their contribution to these processes.

**Figure 2:**
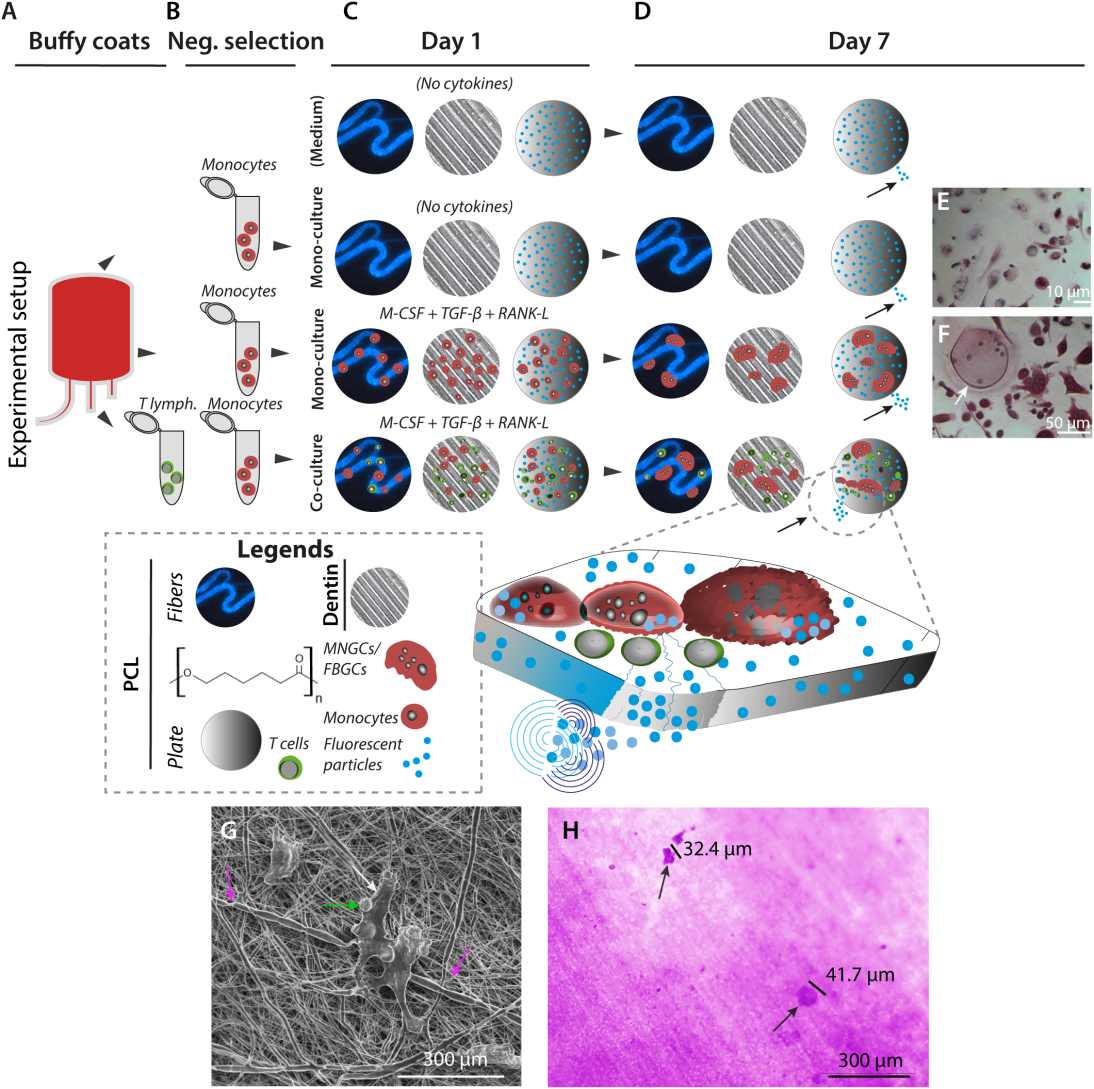
*In vitro* generation of MNGCs on PCL and dentin surfaces. **(A–D)**, MNGCs were generated from primary monocytes in the presence of cytokines on PCL plates with 1-µm embedded fluorescent beads, electro-spun PCL fibers (also with embedded fluorescent 1-µm beads), or human dentin plates. CD14^+^ monocyte precursor cells and allotypic CD3+ T lymphocytes were isolated from human blood donor-derived buffy coats **(A)** by negative selection **(B)**. Immediately following the precursor cell isolation, cell-to-cell fusion was induced by cultivation at a density of 3.5×10^5^ cells/cm^2^ in the presence or absence of M-CSF, TGF-β3, and RANK-L. A co-culture with T lymphocytes in the 2:1 monocyte and cytokines ratio was also prepared. Controls were prepared without cells to monitor the impact of fluorescent beads release from the PCL constructs due to spontaneous PCL hydrolysis **(C)**. After 168 h of culturing, the effect of the incubations was monitored by microscopical imaging and assessment of the release of fluorescent beads (indicated with small black arrows on Day 7) **(D)**. **(E, F)**, Cell morphology at Day 7. TRAP+ non-multinucleated cells were found in the control groups with no addition of cytokines **(E)**. TRAP+ MNGC (white arrow) can be detected for the same cell seeding density in cultures with M-CSF, TGF-β3, and RANK-L cytokines **(F)** on Day 7. **(G, H)** Cellular impact on PCL and dentin surfaces after 168 h of culture. SEM image at high voltage (5 kV) of T lymphocyte-like cells and associated CD14+ precursor cells (green and white arrows) attached to electro-spun PCL fibers with coiling events of the cellular protrusions around single PCL fibers (pink arrows) at magnification ×3,000 **(G)**. Culturing on dentin revealed the resorption capacity of the *in vitro* generated MNGCs by evidence of lacunae formation at magnification ×40 (**(H)**, black arrows).

Peripheral blood mononuclear cells (PBMCs) were harvested from human buffy coats, followed by a negative selection of CD14^+^ cells (**Figure 2A**). As expected, these cells were heterogeneous concerning the CD14 expression, with populations either expressing high or low levels (**Supplementary Figures 2A, B**). Allotypic T lymphocytes (CD4+ and CD8+) were also purified by negative selection (**Figure 2B**). To mimic the clinical use of PCL, constructs resembling suture threads or bioplotted plates as well as autografted bone-like human dentin slices (**Figure 2H**, **Supplementary Figures 2D–G**) were included in side-by-side cultures prepared with either medium alone, pure monocytes, pure monocytes with fusogenic cytokines, or monocytes mixed with T lymphocytes in a ratio of 2:1 (**Figures 2B, D**). Cell-to-cell fusion was induced in the isolated monocyte precursor cultures with the human cytokines: macrophage-colony stimulating factor (M-CSF), transforming growth factor (TGF)-β, and receptor activator of nuclear factor kappa-β-ligand (RANK-L) on Day 1 upon culturing on dentin slices or PCL plate constructs embedding fluorescent 1-μm Glacial Blue™ fluorescent polystyrene beads. Semi-depletion of the culture medium with cytokines was performed daily until termination of the cell culture on Day 7 (168 h) (**Figure 2D**).

Microscopy imaging of monocytes cultured without cytokines (**Figure 2E**) compared to cultures with cytokines (**Figure 2F**) confirmed the successful formation of multinucleated TRAP+ giant cells under the latter conditions. To test these cells’ basic reabsorption and phagocytotic capabilities, side-by-side experiments were done with the cell cultures on PCL or dentin (**Figures 2G, H**). On Day 7 (168 h), the MNGCs coiled around the PCL fibers in close contact with the material (**Supplementary Figure 2C**). The dentin exposed to the cell culture showed lacuna formation (**Figure 2H**, **3A–C**). In addition, the calcitonin receptor-positive mononuclear cell precursors (**Supplementary Figure 2L**) and the generated MNGCs survived even after 504 h under these culture conditions, indicating no *in vitro* toxicity of the PCL constructs (**Figures 4A–M**, **5A–G**, **Supplementary Figures 2H, I**, **Supplementary Figures 5D, E**).

**Figure 3:**
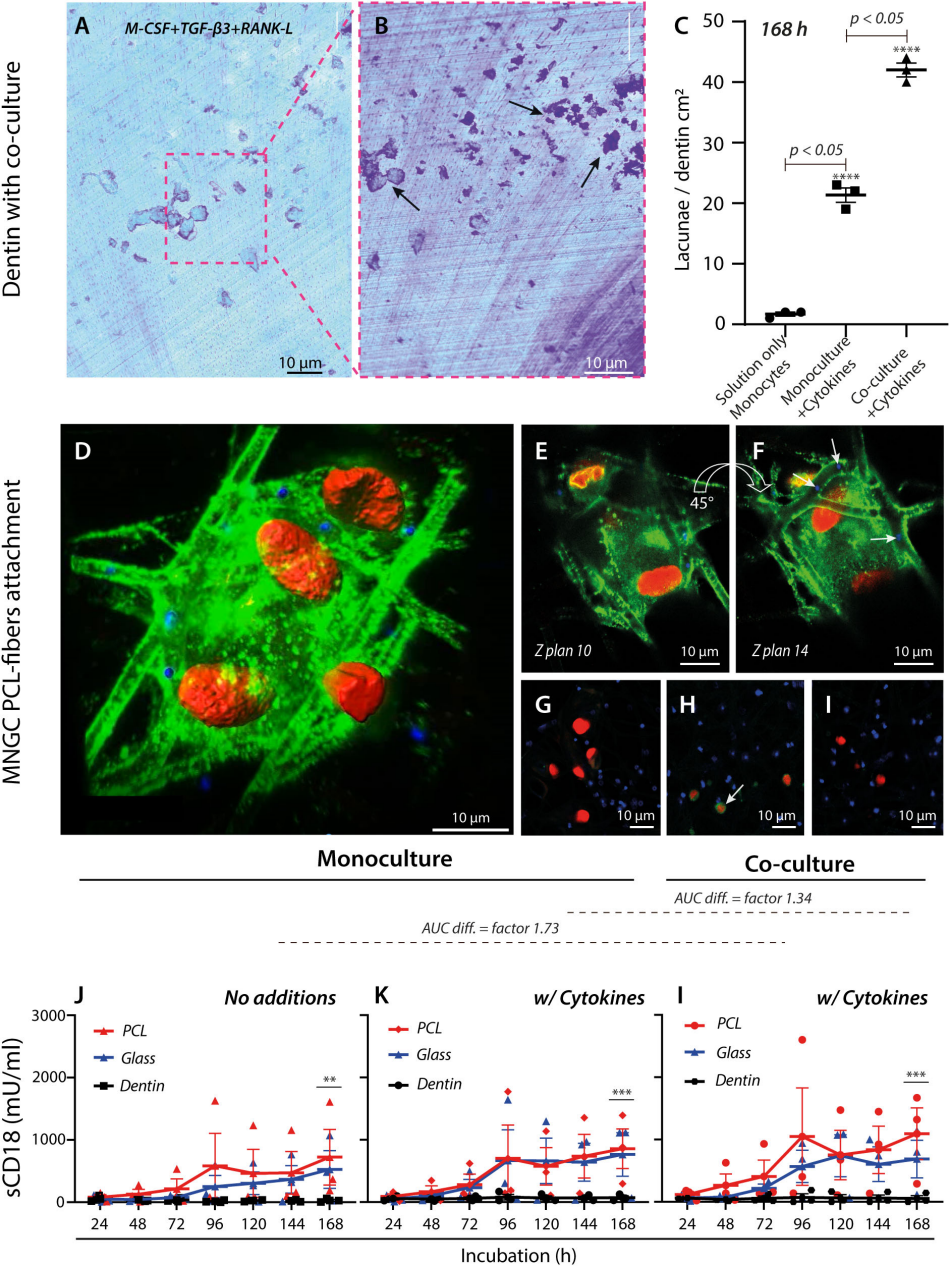
Regulation and adhesion of MNGCs by engineered and natural surfaces. **(A–C)** Quantification of lacunae formation by MNGCs on dentin surfaces. Light microscopy imaging of a Coomassie-stained dentin plate and after co-culture incubation of monocytes and T lymphocytes with the addition of M-CSF, TGF-β3, and RANK-L cytokines **(A)**. Removal of cells by trypsinization on Day 7 revealed lacunae formation (**(B)**, black arrows). Comparison of lacunae formation under conditions without cytokines, with cytokines, and with cytokines and co-culturing of T lymphocytes **(C)**. The assay was made with cells from one donor in 3 independent replicates. **(D–I)**, Confocal imaging (magnification ×20) of a MNGC (plasmalemma) (the star-symbol (*) used is for p-value under 0.05. **** is represented by a p-value <0.0001) attached to electro-spun PCL-fibers carrying shed CD18 integrin (green) with embedded fluorescent beads (in blue, indicated with white arrows **(F)** and cell nuclei stained red. Images are shown at different angles (**(D)**, 45° and in rotations, **(E, F)**. See also **Supplementary Video 1**). Controls were either non-treated specimen **(G)**, with isotype IgG1 primary antibody (**(H)**, white arrow), or secondary antibody **(I)**. **(J–L)**, TRIFMA analysis shows the effect of PCL, glass, and dentin on CD18-integrin shedding over 168 h at three conditions for three donors. Monocytes were seeded at the density for cell fusion, with or without cell fusion-inducing cytokines, or in a cell fusion induction environment co-cultured with T lymphocytes at a ratio of 2:1. The CD18-integrin shedding was compared between conditions without cytokines **(J)**, with M-CSF, TGF-β3, and RANK-L **(K)**, and with cytokines and in co-culture with T lymphocytes. The p-values (after log-transformation) for comparison of the CD18 shedding after 168 h on PCL and dentin surfaces were **p=0.0097 **(J)**, ***p=0.0010 **(K)**, and ***p=0.0008 **(l)** in a two-way ANOVA. The difference in area-under-curve (AUC) for data points between the two materials is shown above the graphs, indicating overall stronger CD18 integrin shedding induced by PCL in the co-culture setup with cytokines **(l)** compared to cultures with only monocytes and no cytokines **(J)**.

**Figure 4:**
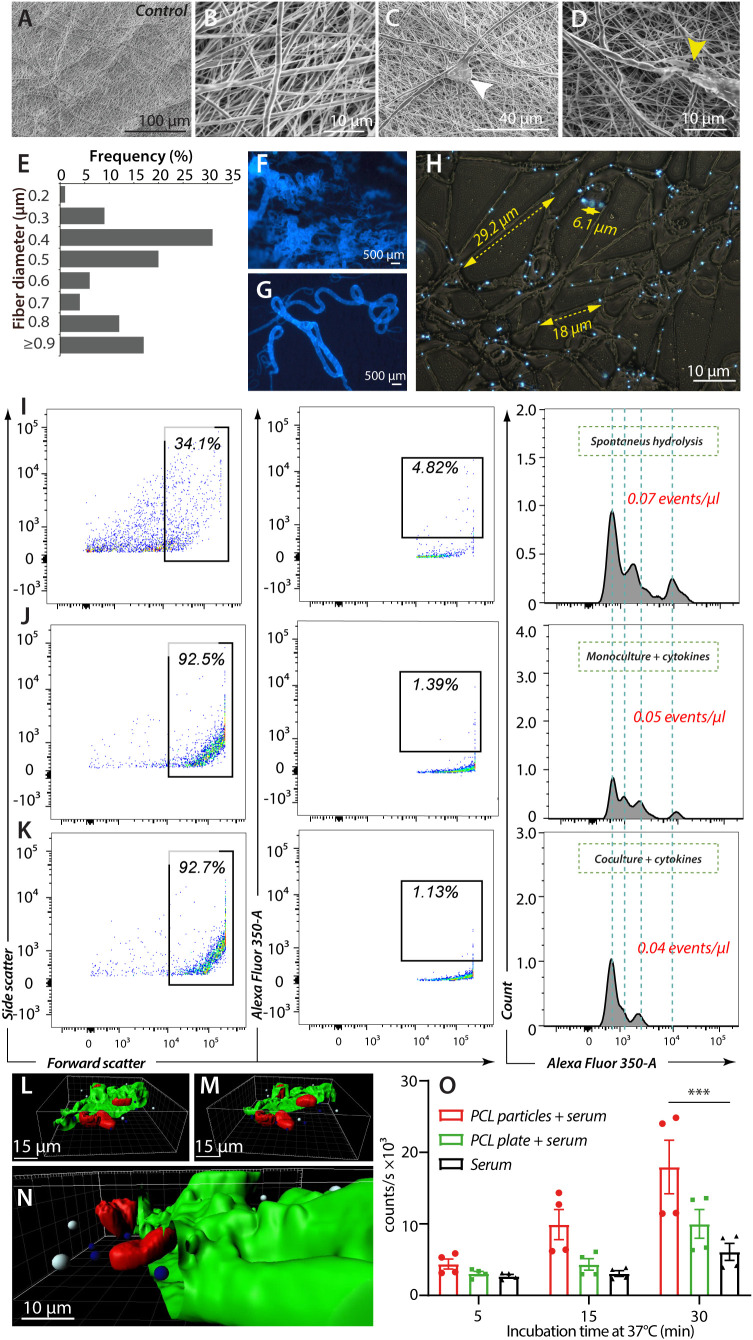
*In vitro* release of fluorescent beads from PCL fibers. **(A–H)**, Morphological characterization of electro-spun PCL fibers with fluorescent beads. **(A, B)**, Control with high-voltage SEM images (5 kV) of electrospun PCL fibers with no prior cell attachment at magnifications ×1000 **(A)** and ×10000 **(B)**. **(C, D)**, PCL fibers after cell attachment (white arrow) at magnification ×3000 **(C)**, and with fiber breakage (yellow arrow) after MNGC co-culture with cytokines and T lymphocytes at magnification ×7500 **(D)**. **(E–G)** Epi-fluorescent imaging of PCL fibers with embedded fluorescent beads at ×10 **(F)** and ×20 **(G)** magnification. **(E)**, Diameters of electro-spun PCL fibers with incorporated fluorescent beads and at the same ratio as shown in **(F–H)**, Distribution of embedded fluorescent beads within electro-spun PCL fibers after washing and before cell exposure. Yellow scale bars indicate typical inter-particle distances within single fibers. **(I–K)**, Flow cytometric analysis of fragment release from electro-spun PCL fibers. The inter-particle distance observed in Panel **(H)**, prompted gating the upper interval of the forward scatter to capture fiber fragments containing one or more beads. The contents of the first gate were analyzed in a plot of fluorescence intensity versus forward scatter (size). A gate was set to enumerate large fragments with robust fluorescence, further plotted as a fluorescence intensity distribution with the total particles calculation. Hatched grey lines indicated shared features of the fluorescence intensity profile between the experiments. **(L–N)**, 3D visualization of a MNGC after 168 h of cultivation with bead-embedded PCL constructs. The CD18-integrin expressing plasmalemma (green) was partly removed by the Zeiss LSM confocal software (see also **Supplementary Video 2**) to demonstrate the multinucleation (red), the phagocytosed beads (blue) and non-phagocytosed bead (white). **(O)** Complement activation at 37°C by equivalent masses of PCL particles or mold-cast plate. Human serum was used as a complement source and incubated for 5, 15, and 30 min. The activation was followed in a standard assay measuring the formation of sMAC. Individual measurements are shown with mean values (columns) and SEM (error bars). Data were analyzed in an ANOVA (n= 4, ***p = 0.0001).

**Figure 5:**
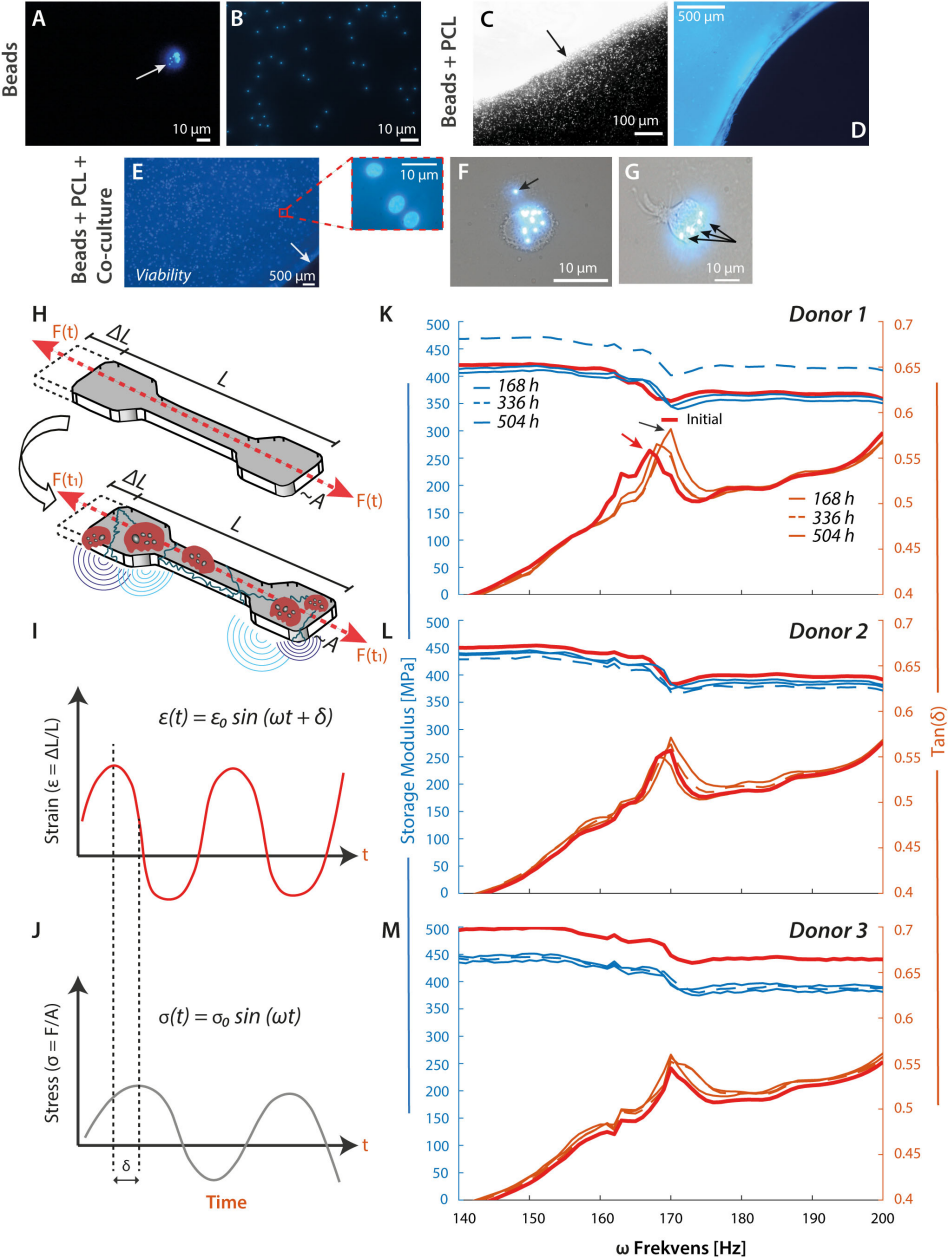
PCL material stiffness increases with OC-like cell exposure. **(A–D)**, Test of cell functionality and longevity of viability. The monocytes were tested for phagocytic capacity before seeding on PCL. The epi-fluorescent microscopic imaging reveals a negative-selected CD14^+^ cell with several internalized fluorescent beads **(A)**, white arrow, and magnification ×20. Appearance of free 1-µm fluorescent beads in culture medium **(B)**. Border of a mold-cast PCL construct with embedded fluorescent beads before washing procedure and cell culture experiments (**(C)**, magnification ×4). The micrograph indicates the diffusion of free fluorescent beads into the medium from the PCL construct (**(C)**, black arrow). The same mold-cast PCL construct (blue) at magnification ×20 and after 3× washing. The embedded fluorescent beads were vaguely visible, while the free beads were quantitatively removed **(D)**. **(E)**, Viability testing of negatively selected monocytes at 3.5×10^5^ cells/cm^2^ on mold-cast PCL and treated with cytokines. The cell nuclei were DAPI stained at Day 7 (magnification ×4). The PCL border is indicated by a white arrow. The red box details three cell nuclei at a random spot, further supported by **Supplementary Figures 2H, I**, **Supplementary Figures 5D, E**. **(F, G)** To test the resorption or phagocytic capacity of the CD14^+^ cells, imaging of engulfed fluorescent beads was probed by epi-fluorescent microscopy. Most particles were found intracellular (**(F, G)**, black arrows), with an occasional occurrence extracellularly. **(H–M)**, Mechanical analysis of mold-cast PCL constructs after cell culture. DMA was performed as illustrated schematically. Elongation of the PCL test specimen by *ΔL* from its original length *L* gives rise to a measurable force *F* **(H)**. A sinusoidal excitation of the PCL test specimen by an amplitude, $\epsilon_{0}$ , and a frequency, ω , characterizes how the elastic energy from the elongation is appreciated by the material. In the dynamic measurement, a phase shift, δ , occurs between the applied strain, $\epsilon \left(t\right)$ **(I)**, and the measured stress, $\sigma \left(t\right)$ **(J)**. Energy is stored temporally and re-released when the material is released during a sinusoidal vibration measured by the Storage Modulus. Energy is lost into the material due to the Loss Modulus, which measures internal vibrations and a temperature increase. Tan $\left(\text{δ}\right)$ is the ratio between storage and loss modulus **(I, J)**. **(K–M)**, Results from DMA of PCL test specimens exposed to monocytes from 3 donors, initial specimen properties against the same specimen incubated in the experiment.

### CD18 integrins are active during MNGC contact with engineered surfaces

The above experiments showed that phagocytic MNGCs could resorb PCL and dentin. We wanted to understand what cell-adhesion mechanisms are responsible for this process.

Initially, the cellular behavior on dentin surfaces was studied. Monocytic precursor cells were either left untreated, treated with cytokines to induce fusion as before (**Figure 2H**), or treated both with cytokines and co-cultured with T lymphocytes (**Figures 3A, B**). After 168 h, a comparison between the three conditions showed a vast increment in the lacunae formation with the addition of cytokines and even further augmentation when T lymphocytes were co-cultured. The interaction of MNGCs with PCL was then considered to determine what adhesion molecules played a role in the contact. The cellular attachment and sCD18 deposition were followed by confocal microscopy imaging by staining both cells and the PCL fiber substrate for CD18 (**Figures 3D–I**, **Supplementary Figures 2H–K**). The confocal imaging revealed an extensive, pseudopodia-driven contact between the cells and constructs with marked deposition of CD18 on the fibers (**Figures 3D–I**, **Supplementary Figure 2J**) in addition to the strong expression of these integrins in the MNGC cell membrane. Furthermore, it was clear that the defects in fibers accompanied the MNGC exposure (**Figures 2G**, **4D**). It strongly implicated the CD18 integrins in MNGC resorption and phagocytosis of PCL.

To make a robust comparison of the engagement of CD18 in contact with PCL and dentin surfaces, the shedding of CD18 was compared with other engineered surfaces, namely glass. PCL plates without fluorescent beads were used as the substrate, resembling the dentin plates included for comparison. Over a course ending on Day 7 (168 h), a steady increase in the sCD18 concentration was observed for the cultures with PCL. For comparing the culture conditions, the area under the curve (AUC) was calculated, with a difference of 1.34 fold between the monoculture of MNGCs and the co-culture of MNGCs and T lymphocytes (**Figures 3J–L**). In contrast, the co-culture differed from untreated monocytes by 1.73 fold (**Figures 3J, L**). The response was similar for the glass surface. Together, the CD18 shedding on the engineered PCL and glass surfaces was only moderately regulated by adding cytokines or T lymphocyte co-culture. By striking contrast, sCD18 was quantitatively undetectable in cultures with dentin. The cytokines or co-culture had no effect on this absence of CD18 shedding. The dentin resorption assay was performed with cells from one of the donors used in the experiment to ensure that dentin resorption occurred. The dentin substrate showed marked lacuna formation, potentiated by monocytic differentiation and T lymphocyte co-culture. This observation indicated that the lack of CD18 engagement in the process was not due to resorption inactivity (**Figure 2H**, **3A–C**, **Supplementary Figures 2D–G**).

### The decay of PCL fibers is not accelerated by leukocyte culture

We wanted a technology that produces PCL constructs with smoother surfaces, permitting a more stringent evaluation of the connection between cellular adhesion and PCL resorption. PCL constructs were made with electrospinning, producing fiber constructs resembling suture threads assembled into a dense mesh (**Figures 4A–D**). The fiber morphology also enabled quantification of the mesh thickness, with 17% of the PCL fibers reaching the same diameter or larger than the 1-µm fluorescent beads (**Figure 4E**), confirming the efficient embedding of the particles. Fluorescent microscopy confirmed a homogeneous distribution of the beads throughout the fibers (**Figures 4F, G**), with a spacing between most of the beads of 5-30 μm (**Figures 4H**). Any defects in the fiber mesh were essentially undetectable (**Figures 4A, B**).

While the microscopic examination revealed evidence of cell-mediated fiber breakage or decay (**Figures 2G**, **4D**), it is hard to translate these data onto a quantitative scale. We used flow cytometry (**Figures 4I–K**) to quantify beads in the supernatants for the culture conditions described (**Figure 2D**) as a proxy of the overall decay of the fibers. From the embedding and spacing of the beads noted above, we expected the emancipated beads to appear in fluorescent fiber fragments of an increased size and lower fluorescent signal than the naked beads. These considerations prompted a gating strategy based on forward scatter, reflecting size, and bead fluorescence to distinguish fiber fragments from cellular debris.

As expected, conditions for bead emancipation in the absence of cells produced a 14%-fraction of fragments with a relatively high forward scatter (**Figure 4I**). Under conditions with cells, this fraction increased was close to 100% (**Figures 4J, K**), however, mainly due to the presence of cellular debris in the supernatant. Identical gates were set for the analyses to exclude the debris, capturing only the highest fluorescence-emitting fragments. Again, the percentage of fragments in the gate confirmed the removal of these beads from cellular debris (**Figures 4J, K**) compared to cell-free conditions (**Figure 4I**). When analyzed in histograms, the fragments revealed spiked populations typically evidencing the inclusion of one or more defined quantum of fluorescence, i.e., beads (48). Moreover, the fluorescence intensity of these spikes was mainly shared between the different conditions, confirming that the particles analyzed had strong similarities concerning the number of fluorescence units included. The number of fragment events per volume of supernatant, i.e., particles in the gates, were also similar. When also taking into account the difference in fluorescence of the particles, essentially a proxy for the amount of emancipated fluorescent beads, there was no evidence that the presence of leukocytes, including MNGCs, to any marked extent, if at all, changed the degradation of the PCL fibers (**Supplementary Figures 3A–E**).

Considering the evidence of cellular adherence and small breaks in the fibers previously noted (**Figures 2G**, **4D**), we asked if the cells could resorb the fragments with particles from the fibers. The uptake of the fragments by MNGCs was analyzed using epifluorescent and confocal microscopy. The imaging showed frequent bead uptake in addition to pseudopodia formation on Day 7 after PCL plate and fiber constructs exposure, including phagocytosis (**Figures 4L–N**, **Supplementary Figures 2H-K** and **Videos 1**, **2**). The intracellular uptake of the beads was studied in confocal microscopy with stains for cell nuclei and CD18 integrins (**Figure 3D**). The beads were located both intracellular, below the plasmalemma, and on the plasmalemma, a picture often seen with particulate material engulfed in CD18-integrin mediated phagocytosis (**Figures 4L–N**). The beads appeared at the anterior cell pole (**Figures 4M, N**), the site of many adhesive processes, including phagocytosis (49).

To better understand the immunological role of fragment release from the PCL constructs, we tested the ability of particulate PCL versus PCL plates formed by melting to activate human complement. During activation of the complement pathways, many functionally active protein fragments are released as part of complement protein cleavage, notably the C3a and C5a anaphylatoxins and chemoattractants. A robust and unified measure involves the formation of soluble membrane-attack complexes (sMAC), which occur at the end of the pathway. When an equal mass of particulate PCL or cast PCL plates was added to conditions permitting complement activation only after 30 min, the sMAC formation by the particles was significantly higher than for serum alone (**Figure 4O**). Although the plate constructs also tended toward complement activation, no significant difference was obtained compared to serum alone. The activation occurred over a reasonably short interval of only 30 min, with a tendency for this order of complement activation to occur after 5 min.

### MNGC resorption increases the mass of the PCL specimen

Next, we investigated the relationship between cell-mediated resorption degradation of PCL and the strength of the material (**Figures 5A–K**).

Young’s modulus is a standard measure of stiffness. Still, procedures for measuring it are typically based on tensile strength, which only changes in materials such as PCL after prolonged exposure to cellular sources. A more sensitive investigation is dynamic mechanical analysis (DMA), where the elongation of a test specimen gives rise to a measurable force (**Figures 5H–J**). A sound wave probes how the material appreciates elastic energy from the elongation from a phase shift, *δ*, in the applied strain, *ϵ(t)*, and in the measured stress, *σ(t)*. The storage modulus measures energy stored temporally during a sinusoidal vibration. In contrast, energy lost to internal vibrations and temperature increase is measured by the loss modulus, with *Tan (δ)* as the ratio between these moduli.

PCL test specimens were cast in a custom-made mold with a dumbbell-like shape, which permitted the bending of the entire specimen without provoking crack formation (**Supplementary Figures 4A–C**). The injection molding formed specimens between 0.20 mm and 0.25 mm in thickness, with essentially the same tensile strength values of 25–30 MPa and Young’s moduli of 240–250 MPa (**Supplementary Figures 4D, E**) corresponding to the previous findings for PCL (50).

In each experiment and before DMA, we ensured that the PCL specimens had no significant artifacts (**Supplementary Figure 5A**). However, microcracks were occasionally visible (**Supplementary Figure 5C**). As a reference, DMA was carried out for each specimen before exposure to culturing conditions and cells. Further, the cell viability was checked (**Supplementary Figures 5D, E**), as well as the resorption capacity (**Supplementary Figure 5H**). Following MNGC exposure, the 0.20 mm thick PCL specimens showed signs of fabricated PCL artifacts and changes, likely to derive from the cellular exposure (**Supplementary Figures 5B, F–H**). Compared to native surfaces (**Supplementary Figure 5B**), quasi-circular openings were easily identified after 168-h of co-cultured MNGC (**Supplementary Figures 5I, J**), suggesting a link to cellular activity as judged by the opening morphology, although in principle, the features could also have originated from the casting process. This observation supported the hypothesis that cellular adhesion would act to decrease PCL material strength and stiffness.

The DMA produced curves with phase shifts in *Tan (δ)* for ω values at ~170 Hz compared to the DMA of the (initial) PCL specimen before incubation. For incubations with cells, the negative peak in *Tan (δ)* was right-shifted towards higher ω values, at least for the incubations of 168 h (**Figures 5K–M**). This was unlike the response for the PCL specimen kept under culturing conditions but without cells, where the ω value was left shifted to lower values compared with the untreated PCL specimen (**Supplementary Figure 6**). Further cellular incubations for 336 h and 504 h reduced ω value towards the value for the untreated specimen (**Figures 5A–K**). The noticeable right shifts in storage moduli suggested the addition of a PCL-associated mass. In contrast, the later left shift in storage moduli suggested a loss. The difference between experiments with and without cells pointed to at least two types of processes involved: one, noticeable from the early stages of cell incubation, increasing the mass of the specimen, and another, noticeable without the cells, reducing the mass.

## Discussion

Advanced implant materials will likely significantly change the possibilities of restorative surgery. In this venue, PCL is a promising choice of material because of its relatively benign integration with body tissues and the several valuable aspects of casting and resulting properties of the graft. However, a significant inflammatory response can be triggered like for many other engineered graft materials. Significant macrophage-driven degradation of PCL, as part of the innate immunity to PCL, has been revealed, and reactive oxygen species and hydrolytic enzymes generate the degradation. The oxidative degradation is dependent on the PCL microarchitecture. PCL is degradable, and in another recent study, PCL-based platelet particles were shown to be completely metabolized by macrophages through lysosomal degradation (51, 52).

Here, we compared *in vivo* data on the recruitment of leukocytes into the graft site with *in vitro* experiments on natural and engineered surfaces. Our findings depict a surprising role of both immature myeloid cells and OC-like MNGCs in integrin-mediated contact between the PCL grafts. Unlike previous assumptions from the microscopic indents made by these cells in both natural and PCL graft surfaces, our quantitative assays show the cell-mediated degradation to be relatively modest compared to spontaneous hydrolysis. By contrast, from mechanical analysis after cell culture, the monocytes and the MNGCs appear to remodel the PCL, strengthening the material. These findings lead to new insights into the immune system’s role in maintaining graft functionality.

Our *in vivo* experiments determined a previously unappreciated PCL association with non-TRAP positive MNGCs because of this association, sometimes referred to as FBGCs. Interestingly, we observed an additional infiltration of T lymphocytes in some grafts. It agrees with other *in vivo* studies, revealing an increased presence of PCL-associated T lymphocytes (7). Our findings are also supported by studies showing that the FBGCs are generated *in vivo* by different pathways than regular OCs (53–56). Although sharing the monocytic precursor origin, these MNGC-variants differ in their interactions with other cell types from OCs. For instance, FBGC formation is unaffected without T lymphocytes (56, 57). We used blinded targeting for our cell counting. Despite the non-significant TRAP+ MNGC-formation *in vivo*, certain PCL areas were rich in TRAP+ cells. From the literature, we speculate that small sections of the microenvironments enable IL-4-induced differentiation of recruited precursors to the PCL graft site to TRAP+ FBGCs or by maturation of other types of MNGCs capable of *de novo* TRAP synthesis (56). The low number of *in vivo* TRAP+ cells, adjoining the PCL implants and at the skull tissue interface, could indicate the involvement of subclasses of MNGCs. We suggest our findings include multinucleated FBGCs. Previous research supports this notion. The known *in vivo* subsets are referred to as pro-inflammatory M1-like MNGCs and anti-inflammatory, wound-healing M2-like MNGCs. In these cells, a reduced TRAP, calcitonin receptor, and matrix metalloproteinase (MMP)-9 expression have been found when compared to OCs. Furthermore, they can maintain long-term bone volume and possibly contribute to tissue regeneration. Still, it remains unclear precisely what conditions guide the precursor cells to fuse and form these subtypes of FBGCs (58–60). Other striking differences in the biological behavior between OCs and FBGCs include that OCs form basolateral ruffled border in connection with the underlying surfaces. Only OCs reabsorb bone. However, FBGCcs can demineralize superficial areas on bone surfaces (61).

Following our observations *in vivo*, we designed several *in vitro* experiments to understand leukocytes’ impact on PCL. Comparisons were made to more natural surfaces, namely dentin. Glass was another engineered material. As cellular sources, we used a simple culture of human monocytes or monocytes cultured with cytokines to maturate these cells into OC-like MNGCs. Further, as a follow-up on the T cell infiltration *in vivo*, we also made co-cultures of allotypic T lymphocytes. We first deselected non-OC progenitors from the heterogeneous cell pool of the buffy coats. By this approach, only monocytes were left in the culture or seeded onto PCL constructs at a density and with cytokines suitable for cell fusion.

Additionally, cultures with T lymphocytes were added in a ratio of 2 T lymphocytes per monocyte to mimic the role of T lymphocytes in osteoclastogenesis (57). The cell ratio was chosen to permit long-term cultures. Since all subsets of T lymphocytes are known to influence OC formation, no efforts were invested in further separating these cells (62).

We wanted to detail the interaction between the cultivated cells and their adhesion substrates at the functional and molecular levels. Exposure of the three types of cultures to dentin surfaces found a clear difference, with OC-like MNGCs outperforming the monocytic precursors concerning lacunae formation. As expected from the literature, the role of T lymphocytes in the OC-formation (56) was also confirmed by the marked increase in lacunae formation when these cells were added. We imaged cells in contact with electro-spun PCL fibers to obtain a practical microscopic analysis of the interaction between PCL and the MNGCs. Among the adhesion molecules likely to play a role in such contact, we analyzed the involvement of CD18 integrins. These molecules have previously been shown to interact with polymeric surfaces (37, 41) through a combination of broad ligand binding specificities, i.e. especially CD11b/CD18 and CD11c/CD18 (63) and the ability of engineered surfaces to accommodate the deposition of soluble proteins ubiquitous in cell cultures (42, 64). By confocal imaging, we showed an intense CD18 deposition on PCL fibers after seven days. The OC-like cells also seemed able to phagocytose the fluorescent beads embedded within the fibers, verified by both 2D and 3D imaging. These images indicate a more mechanistically complex PCL degradation than only through a phagocytotic process of already emancipated fragments, i.e., from spontaneous hydrolysis.

The marked deposition of CD18 on the PCL fibers is entirely consistent with earlier observations on the enzymatic shedding of these receptors. Shedding is linked with activity in adhesion and migration (65–69) and there is evidence that CD18 remains bound to natural ligands such as ICAM-1 even after shedding (65, 66, 69, 70). These mechanisms probably act as a release mechanism that enables cellular de-adhesion from substrates, enabling cellular migration (67, 69). We measured sCD18 in supernatants from the cell cultures on cast PCL, glass, or dentin surfaces. On PCL and glass surfaces, the sCD18 concentration increased time-dependently. Still, there was no major influence from adding cytokines or T lymphocytes. By contrast, no sCD18 release was observed on dentin surfaces, probably excluding CD18 integrins as part of lacunae formation by OCs. This finding points to the engineered surfaces as recognized by the leukocytic adhesion mechanisms in a fundamentally different way than natural bone surfaces, and it identifies CD18 integrins as central in the inflammatory response to these materials.

The degradation of PCL may follow two different pathways in our experiments, namely spontaneous hydrolysis or catalyzing by the many proteolytic enzymes produced by myeloid cells. The simple geometry of electro-spun fibers, together with a chemistry of the fluorescent polystyrene beads inert to esterases, predicts that fragments with high fluorescence, *i.e.*, containing multiple beads, must be relatively large at approximately ~10 µm. This rationale enabled a gating strategy excluding cellular debris, contributing to the counting of emancipated fluorescence. Surprisingly, despite the microscopic evidence of indents made by the cells in the PCL constructs, the cellular cultures did not affect the emancipation of fluorescent fragments. It was kept at a level close to that found for spontaneous hydrolysis, questioning if the monocytes or OC-like MNGCs are a major source of PCL decay. Taken together with the microscopic analyses, showing cells in remarkably tight and controlled contact with the PCL fibers, the cell-mediated contacts may produce localized effects but not major degradation. A limitation in interpreting these experiments is that the *in vivo* hydrolysis and the results obtained *in vitro* could quantitatively differ. Our conditions mimic certain physiological conditions, *e.g.*, standard culture media and a stabilized physiologic pH. Even so, the more complex conditions *in vivo* could affect hydrolysis and enhance the function of PCL-degrading cells. As already noted (59, 60), these conditions include MMPs. Several esterases, but typically not MMPs, may directly degrade the PCL (71). Still, at least on speculative grounds, the high activity of many MMPs could act indirectly to clear away protein deposits on the PCL surface. In this way, access of esterases to the PCL chemical bonds would be promoted, in turn facilitating PCL degradation.

Nevertheless, spontaneous hydrolysis contributes to inflammation. PCL fragments are potent activators of the complement system, and more so than for intact plates, probably driven by the increased surface area and altered topology such curvature (72). In the process, the production of the anaphylatoxins C3a and, especially, C5a support chemotaxis of leukocytes into the vicinity of PCL implants and dilation of blood vessels, all adding to the inflammatory reaction surrounding the implants.

Since the CD18 integrin-mediated contact and localized degradation by the monocytes and the MNGCs in our experiments were not quantitatively responsible for PCL construct degradation, we used mechanical analysis of the constructs to probe for structural changes induced by the cells. We investigated such changes in a time frame of maximally three weeks, expecting the material stiffness to decrease from the cellular indents. Indeed, others found a reduction in Young’s modulus correlating with a loss of mass albeit in processes proceeding over months (50). These measurements were conducted in a static tensile test with a single pull, conducted until the specimen broke. We performed the same type of experiments on tensile strength but with no significant changes in Young’s modulus or the mass of the cultured specimen. A more sensitive test was then applied using DMA, providing information on changes in the materials’ moduli from 0–200 Hz. Noticeable shifts in moduli were observed around frequencies of 170 Hz. Translation of these results to simpler terms means that PCL time-dependently gained mass and stiffness, at least in the early stages of cell exposure. Evidence of these changes has been scantly reported before (4, 50), but not resolved on the short time scale now reported here. Control experiments without cells indicated the involvement of another process reducing specimen mass, which likely relates to hydrolysis, as noted in the flow cytometric analysis. Our experiments documented a protein deposition from the attachment matrix, which is exemplified by the CD18 integrins. However, our experiments do not imply that CD18 integrins are the only part of such proteins from the MNGCs deposited on the PCL surface, nor are they necessarily quantitatively causing the strengthening of the material. From other literature, one may speculate that additional adhesion molecules, such as the integrins α_v_β_3_ and α_5_β_1_ as well as their ligand fibronectin, also contribute to the deposition (73). These molecules may account for the cell-dependent mass increase recorded in DMA. Our findings are evidence that important adhesion molecules are deposited as an integral part of the contact with PCL, providing insight into the type of molecular mechanisms responsible for the strengthening. Once the cellular deposition is attenuated after approximately three weeks of *in vitro* culture, as monitored by the diminished release of sCD18, the spontaneous hydrolysis starts to dominate, and mass is lost from the PCL specimen.

**Figure 6:**
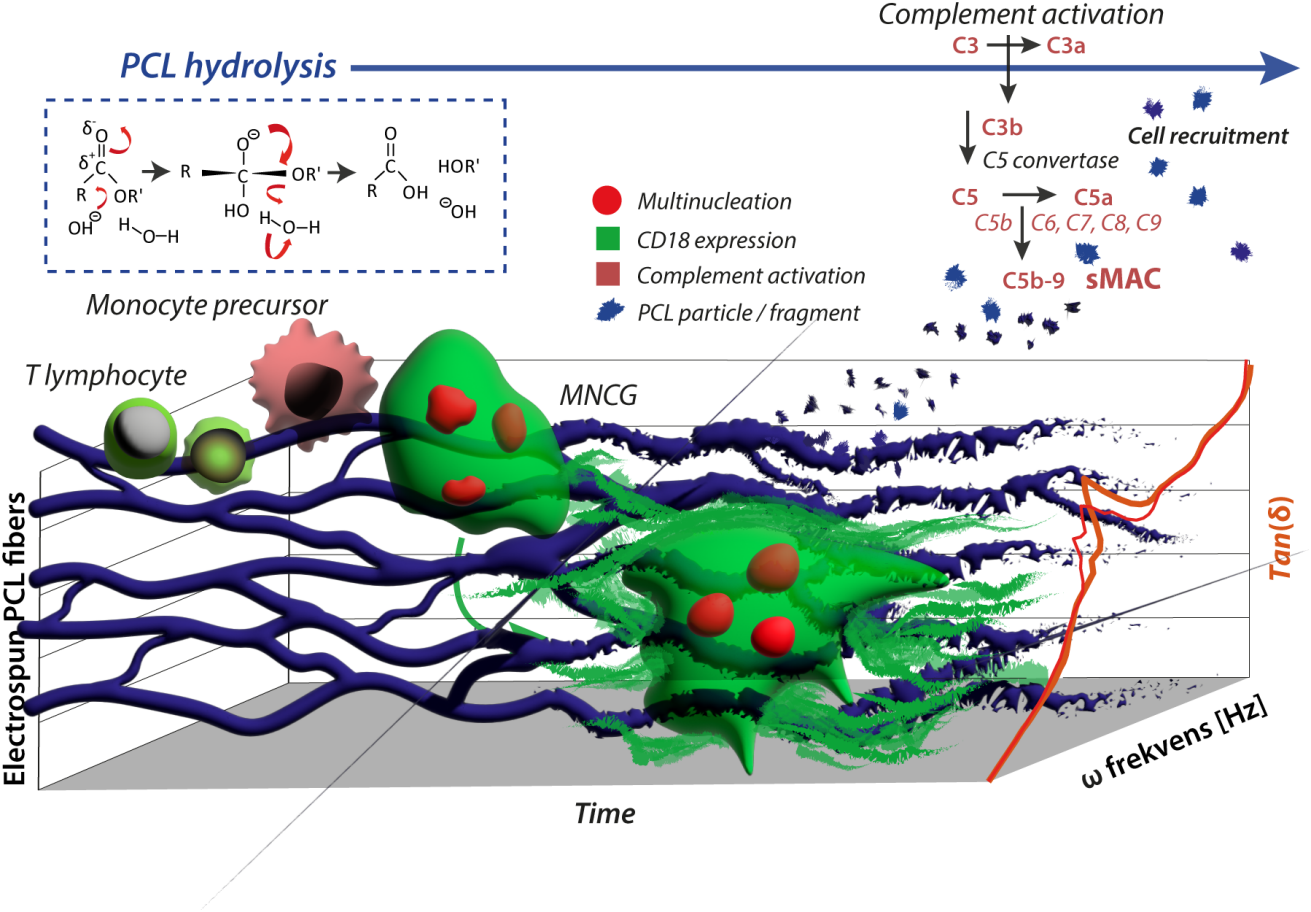
Schematic presentation of the immunostimulatory effect of PCL. Adhesion of monocytes on electro-spun PCL fibers with embedded fluorescent particles is indicated. In a fusogenic environment, MNGCs were formed *in vitro* from monocytic precursors resembling the observations from grafts *in vivo*, where monocytes transmigrate into the graft lesion and maturate into FBGCs. Flow cytometry shows that the release of PCL particles from the fibers is not increased by the presence of MNGCs compared to the spontaneous hydrolysis of PCL alone. However, the particulate PCL triggers complement activation (sMAC), contributing to leukocyte recruitment to the graft site. The MNGCs were firmly CD18 integrin positive. The staining found the deposition of shed sCD18 in the adhesion matrix on the PCL constructs. In effect, this explains at least in part the reinforcement of the PCL grafts, revealed in DMA by increased Tan(δ) values (right shift).

Our study provides a significantly changed picture of the immunological consequences of PCL graft cranioplasty (**Figure 6**) and maybe other *in vivo* applications of PCL as well. Unlike the focus on FBCG formation in response to grafts, our experiments *in vivo* show a strong involvement of TRAP-, immature monocytic precursors invading the graft site. While these cells are relatively inactive towards natural bone, in our *in vitro* assays, their response to engineered surfaces such as PCL and glass was undisguisable from that of more maturated cells, even in co-culture with T lymphocytes. The role of monocytic infiltration appears to relate to a kind of repair process, strengthening, rather than degrading, the PCL. As such, the monocyte involvement is reminiscent of what has been described for diseases with a neuroinflammatory component, such as mild-cognitive impairment (40), often progressing to Alzheimer’s Disease. In these diseases, early stages of monocyte recruitment to the inflamed tissue, *i.e.*, the brain, appear to involve a healing aspect by debris clearance (40), which then, if the disease progresses also may become a source of tissue destruction, aggravating symptoms (74). We suggest that the role of the peripheral immune system in PCL graft responses is equally double-edged, with early stages characterized by the recruitment of multiple sets of leukocytes and attempts to heal the lesion. In later stages, the inflammatory response may grow and cause unwanted effects with overt damage to the graft. This model may help guide therapeutic intervention, advancing the immune-mediated healing of graft lesions and dampening function-destroying influences.

## Methods

### Ethics in animal and human studies

All animal experiments for this study were conducted according to the Aarhus University Animal Care and Use Committee and the animal welfare regulations approved by the Danish Animal Research Inspectorate and conformed to Danish law (application no. 2012-15-2934-00362). The use of human caries-free third molars for dentin plate assay was approved by the Central Denmark Region Committee on Biomedical Research Ethics. Buffy coats were obtained from healthy donor blood collected by The Blood Bank at Aarhus University Hospital in an ethically approved protocol (Scientific Ethical Committee for the Mid-Jutland Region, permission number 77).

### Animal experiments and sample analysis

Anesthesia, surgical procedures, and post-operative treatment performed on three animals have been described elsewhere (75). Briefly, samples were collected from eight-month-old female Danish Landrace pigs (*Sus scrofa domesticus*) as described earlier (75). Two non-penetrating calvarial bone defects were created in each pig, positioned 10 mm apart using a cannulated drill bit. In one defect, a cylinder-shaped PCL scaffold of 10×10 mm was inserted. In the paired defect, autograft bone, gained from the skull trepanation, was inserted as control. The animals were treated postoperatively with 1 ml/10 kg Streptocillin™ (Boehringer Ingelheim, Ingelheim, Germany) for three days. The PCL scaffold implantation time was eight weeks. Afterward, animals were euthanized with phenobarbital overdose. The calvarial bone was resected and kept at -80˚C, then cut in half, dehydrated in ethanol (70%–99%), and embedded in methylmethacrylate (MMA). A cylindrical mold containing the implant or empty defect was extracted and sectioned parallel and 1 mm offset to its vertical axis using a microtome (Polycut E; Reichert-Jung, Heidelberg, Germany).

The 7-µm thick calvarial biopsies were gained from two levels in the sagittal plane with 400 µm between each level using a microtome (Polycut E™; Reichert-Jung, Heidelberg, Germany). The samples were stained with GT, as mentioned above. Immunohistology was performed to investigate T lymphocyte infiltration at the cranioplasty side for separate blinded chosen biopsies. The CD3+ cell infiltrates after eight weeks were compared between the autograft and PCL scaffold sides. After microtomy, the 7-µm sections were deacrylated with 2-methoxy-ethyl acetate (Merck) for 10 min. This step was followed by rehydration in graded alcohol series of 100% (v/v), 96%, 75%, 50%, and 20% for 5 min each, and then thoroughly washed with distilled water. The samples were then incubated in citrate buffer (pH 6.0) for 10 min, treated with microwaves (1000 W) for 20 min, and allowed to cool at RT for 30 min. Blocking was made in 1% (w/v) BSA for 30 min. at RT. After a rinse in Tris-buffered saline (TBS; pH 7.6; 0.05 M) with 0.03% (w/v) Tween20, the primary polyclonal rabbit antibody to CD3 (#C7930, Sigma) was diluted 1:200 and applied for 60 min, at RT in wet and dark chamber. This second to last step was followed by washing x 4 in Tris-buffered saline (TBS; pH 7.6; 0.05 M) containing Tween 20^®^ (0.03%). Secondary staining, using Alexa Flour™-488 goat anti-rabbit IgG diluted 1:500 PBS (Thermo Fisher Scientific), was done for 60 min in a wet dark chamber at RT. The samples were washed four times in PBS and then rinsed in distilled water. Three negative controls were prepared for the experiment, namely *i)* incubation with 5 µg/ml rabbit IgG1 exposure, *ii)* incubation with secondary goat-anti-rabbit antibody alone, and *iii)* no incubation with antibody. Before confocal imaging, all samples were sealed with cover slides on biopsy glasses (Thermo Fisher Scientific) mounted in fluorescence medium (Dako, Glostrup, Denmark) and stored in the dark at 4°C before confocal imaging and quantification. Further steps for both *in vivo* and *in vitro* samples were carried out by using Olympus IX-83 fluorescent microscope with an LSM710 Zeiss Confocal with 63×/1.30 NA water objective lens, using Zen software (Zeiss). Multichannel images were processed using Imaris 8.2 software (Oxford Instruments, Tubney Woods, Abingdon, UK). Brightness and contrast adjustments were applied for the channel independently.

The same TRAP staining protocol was followed, as listed for the *in vitro* TRAP staining approach. Goldner’s trichrome staining of the specimens was performed as previously described (76). MNGCs and lacunae were counted using the newCAST software (version 34 3.4.1.0; Visiopharm A/S, Hoersholm, Denmark).

### Manufacture of PCL constructs

Compact plastic plates for cell culture experiments were made from polycaprolactone (PCL, Perstorp, UK) polymers with a molecular weight of 50 kDa. The fabrication was performed using a BioScaffolder™ 3.2, GeSiM (Radeberg, Germany). Plates were built with layered deposition of polymer strands by extruding molten PCL from an extrusion die with an inner diameter of 200 mm. To increase surface hydrophilicity, the PCL plates were treated with 5 M NaOH for 3 h, neutralized with PBS, and rinsed in sterile water. The PCL plates were then disinfected in 70% (v/v) ethanol for 24 h and dried in a sterile desiccator for 3 days. The PCL plates were then characterized using scanning electron microscopy (SEM) (Nova NanoSEM 600; FEI Company, Eindhoven, The Netherlands).

For plate constructs with homogeneous incorporation of Glacial Blue™ beads (#FC03F; Bangs Laboratories Inc, IN), 1.5 g of dry, 50 kDa PCL particles were mixed with 0.75 g (750 µl) beads by vortexing for 1 min. The composition was then heated to 100°C for 20 min. The melted composition was placed on a borosilicate glass plate (Sigma-Aldrich, LA). After cooling at room temperature for 24 h, the samples for the Glacial Blue beads release and phagocytosis essay were obtained using a sterile biopsy punch (Acuderm, Fort Lauderdale, FL) with an inner diameter of 7 mm. The punched PCL samples were disinfected in 70% (v/v) ethanol for 24 h and treated for 3 h in 5 M NaOH in a laminar flow bench. Before cell seeding, the samples were rinsed twice in PBS and dried in 24-well Corning^®^ Costar^®^ cell culture plates (Sigma-Aldrich, St. Louis, MO) in a sterile desiccator for 3 days.

The material composition of the electro-spun PCL fiber constructs was a 30% (w/v) PCL solution (Capa™ 6500, 50 kDa; Perstorp, UK) in 2,2,2-trifluoroethanol (TFE) (#91690; Fluka™, Honeywell Research Chemicals, Charlotte, NC) and stirred overnight. This step was followed by adding 50 µl Glacial Blue™ beads (#FC03F; Bangs Laboratories Inc, IN) to the solution (1 ml volume in total) and stirring for another 5 h. The suspension was mounted in a 3-ml syringe fitted with a metallic needle of 0.9-mm inner diameter. The syringe was fixed horizontally on the syringe pump (#AL-1000-220Z, World Precision Instruments, Sarasota, FL), and an electrode of high-voltage power supply (Gamma High Voltage Research, Ormond Beach, FL) was clamped to the metal needle tip. The flow rate of the polymer solution was at 1 ml/h, and the applied voltage was at 12 kV. The tip-to-collector distance was set to 15 cm. A grounded rotating rim collector (r = 5.1 cm, w = 7.3 cm) covered by a clean aluminum foil was used for the fiber collection with a rotary speed of approximately 100 RPM. The ambient temperature was 21°C, humidity ranged from 30% to 60%, and the spinning time was 1 h. The electro-spun fibers were dried overnight under a vacuum (Freezone Triad, Labconco, Kansas City, MO) to remove excess solvents. The dried fibers were soaked in 0.1 mM NaOH for 1 h to increase surface hydrophilicity, followed by rinse in PBS. Afterward, the PCL fibers were treated overnight in the freeze drier. The approximately 1-mm thick electro-spun PCL fiber membranes (2 cm in diameter) were stored in the dark at 4˚C before *in vitro* cell experiments. Samples were then punched out from the membranes using a sterile biopsy punch (Acuderm) with an inner diameter of 7 mm. The punched PCL samples were disinfected with 70% (v/v) ethanol treatment for 1 h, rinsed twice in PBS, and air dried in 24 wells of Corning^®^ Costar^®^ cell culture plates (Sigma) before cell seeding. The fiber morphology was examined by high-resolution scanning electron microscopy (FEI, Nova 600 NanoSEM) at 5 kV. The fibers were placed directly into the SEM chamber without metal sputtering or coating.

The three-dimensional grid PCL structures for the *in vivo* experiments have been carefully described elsewhere (75). They were constructed by extruding the PCL from a needle with the same diameter (inner diameter of 200 µm and final fiber diameter ~175 µm) in a layer-by-layer deposition with a 600 mm/min deposition speed. The distances between fibers were ~1000 µm (0°/105° pattern) to allow for a high porosity. The PCL constructs were cylinder-shaped (10×10 mm) and sterilized in a vacuum chamber using ethanol concentrations of 96%, 70%, and 50% (v/v) for 30 min at each concentration, followed by rinsing in sterile water. As for the *in vitro* setups described, the cranioplasty constructs were treated with 1.25 M NaOH for 16 h to increase surface hydrophilicity, then neutralized in 1 M HCl for 1 h, neutralized with PBS, and rinsed in sterile water.

To manufacture PCL plates for DMA by applying oscillation forces, a mold (**Supplementary Figures 4A–C**) was made to cast dumbbell-like PCL specimens, following the ASTM International, PA, D638–10 standard. The geometric specifications were applied using SolidWorks 2010 (Dassault Systems, Paris, France). The setup was prepared by machining a T7050 aluminum block as received (Hydro, Tønder, Denmark). To meet the test specimen-specific dimensions, the mold was composed of aluminum (male-to-female molding concept) with ventilation holes to escape trapped air during fabrication. A phase separation for each sample (0.09 g/specimen of 50 kDa PCL granules) was induced by a heating/cooling cycle, i.e., melting of PCL granules at 100°C for 15 min., followed by a cooling step at 50°C for 45 min. The design included an M5 bolt, which served to manually push the piston that injected molten PCL at 80°C into the cavity. PCL from Perstorp (UK) was used as above.

### Purification of human leukocytes and maturation of MNGCs

Peripheral blood mononuclear cells (PBMCs) were isolated from 0.9% NaCl four times diluted leucocyte-rich buffy coats from healthy donors by two-step density centrifugation to reduce the number of thrombocytes, using sucrose polymer Ficoll Paque PLUS (Amersham Pharmacia Biotech AB, Uppsala, Sweden). Initially, cells were centrifuged for 20 min at 20°C (180×*g*), followed by a collection of the plasma phase, discarded, and an additional centrifugation step for 20 min at 380×*g* was performed. PMNCs from the interphase were carefully harvested, washed twice with 4°C cold PBS containing 1 mM EDTA, counted using a hemocytometer, and kept at 4°C until further processing or cryopreserved at -135°C until later use. CD14-positive monocytes and CD3-positive T cells were isolated by negative selection, using Dynabeads^®^ Untouched™ Human Monocytes kit (#113.50D, Life Technologies, Paisley, UK) and Dynabeads^®^ Untouched™ Human T cell kit (#113.44D, Life Technologies, Paisley, UK) respectively, according to the manufacturer’s instructions. The purity and viability were >90%.

Differentiation of monocytes to macrophage-like cells and further maturation of MNGCs to OCs-like cells was performed (77). Cells were cultured for seven days in a cytokine environment containing 25 ng/ml macrophage colony-stimulating factor (M-CSF, #PHC2044; Invitrogen, Carlsbad, CA), 40 ng/ml receptor activator of nuclear factor kappa-B ligand (RANK-L; PHP0034, Invitrogen, Carlsbad, CA), and 5 ng/ml recombinant human transforming growth factor beta 3 (TGF-β3; #243-B3, R&D Systems, Minneapolis, MN) dissolved in alpha minimum essential medium (αMEM) without nucleosides (22561-021, Invitrogen, Carlsbad, CA) and 10% (v/v) fetal calf serum (FCS; Biochrom AG, Berlin, Germany) with a precursor cell density of 3.5×10^5^ cells/cm^2^. Cells were cultivated either in monoculture (monocytes) or in co-culture with T lymphocytes in the ratio of 1:2 in a humidified atmosphere with 5% CO_2_ at 37°C. The cultured cells were re-fed every second day by semi-depletion.

### MNGC degradation of PCL and dentin plates

Following seven days of culturing, the *in vitro* generated MNGCs in 24-well culture plates were washed twice in tris-buffered saline (TBS), pH 7.6, and fixed in 70% (v/v) alcohol. Cells were then washed for 3×5 min in distilled water and kept in the dark for 1 h before staining with Mayer’s Hematoxylin for 5 s. Following a second wash of 3×5 min in distilled water, all samples were mounted in glycerol. Tartrate-resistant acid phosphatase (TRAP) enzymatic staining was performed by adding 0.6 ml of 4% (w/v) pararosanilin with 2 M HCl (Solution B; #N2125, Sigma) to 0.6 ml of 4% sodium nitrite (1.06549, Merck). This solution was carefully mixed. After 60 s, 450 mg of wine acid and 60 ml Michaelis buffer (#1.00804, Merck) were added. The pH value was then adjusted to 5.0-5.1 with 75 drops of 2 M NaOH (TitriPUR™, Merck). Subsequently, a second solution was made by mixing 2 ml of N, N-dimethylformamide (#3034, Merck, Darmstadt, Germany) with 20 mg of naphtol-as-bi-phosphat (Solution A; #N2125, Sigma). The 2 ml solution was mixed with the primary solution, followed by filtration. Slides were incubated within solutions A and B for 30 min and washed in distilled water. The cells were counted using a light microscope (Eclipse 80i™; Nikon, Tokyo, Japan) equipped with a motorized Proscan 11 stage (Prio, Rockland, MA), a MT1201 microrater (Heidenhain, Traunreut, Germany), a DP72 camcorder (Olympus, Tokyo, Japan). Image analysis was made with the newCAST™ software (version 34 3.4.1.0; Visiopharm A/S, Hoersholm, Denmark), displaying an unbiased counting frame and sampling the culture slides systematically and randomly. Cells with osteoclast-like phenotype were defined as TRAP-positive cells with three or more nuclei.

Human caries-free third molars were obtained and stored in minimum essential media (Gibco Life Technologies™; Thermo Fischer, Waltham, MA) for less than 2 weeks before sectioning. The teeth were mounted onto plexiglas slides with Technovit 4000 (Exakt Technologies, Inc., Oklahoma City, OK) and hereafter cut perpendicular to the long axis of the root into 150-μm thick discs using a precision saw (#3031; Exakt). The discs were rinsed in PBS and sterilized by gamma radiation (173 krad per tooth) using a Gammacelle 2000 (Atomenergikommissionen, Risø, Denmark) (78). The radiation dose at the chamber’s center equaled 1.65 Gy with an exposure time per 150-μm dentin plate of 162 s. Before use in cell cultures, the discs were placed in 70% (v/v) ethanol for 3 h and washed four times in PBS. To study lacunae formation after cell culture, the dentin plates were washed twice in PBS and treated with 1 M sodium hypochlorite solution for 10 min, followed by sonication (Bioruptor™; Diagenode, Seraign, Belgium) in 1 M hypochlorite for 10 min and distilled water for another 10 min. The discs were then washed thoroughly four times in distilled water, air dried, and stained by using Coomassie Brilliant Blue™ for 30 s, rinsed in distilled water for 1 h, and air dried before light microscopy and evaluation with the newCAST software as above.

### Adhesion and degradation of electro-spun PCL fibers by MNGCs

Monocytes at the density 3.5×10^5^ cells/cm^2^ were exposed to the electro-spun PCL fibers on Day 1 under sterile conditions. MNGCs formation was induced with cytokine treatment as above. The MEM culture medium was semi-depleted and replenished every second day. After seven days of culturing, cells were washed twice in PBS for 5–10 min per washing step. This was followed by fixation in 3.7% (v/v) paraformaldehyde for 10 min at room temperature. After fixation, the cells were washed twice in PBS and blocked for 30 min at room temperature in 1% (w/v) BSA and mouse IgG (100 µg/ml) dissolved in PBS. Following prior titration of the KIM127 and KIM185 antibodies (custom-made from American Tissue Culture Collection’s hybridomas CRL2838 and CRL2839 by GenScript, Piscataway, NJ) to human CD18, these were diluted to 5 µg/ml and added into the samples separately. At this step, isotype mouse IgG1 (MA5-14453, Thermo Fisher Scientific), also at 5 µg/ml, or secondary antibody (Thermo Fisher Scientific) alone applied in a dilution of 1:500, were used as negative controls and incubated for 24 h at 4°C. This step was followed by four washes in PBS for 4–10 min. After the four washes in PBS, cell nuclei were stained with RedDot™2 (Biotium Inc, Freemont, CA) by incubation for 30 min at RT. All samples were washed twice in PBS and rinsed twice in distilled water before placement of the PCL constructs with adhered cells on Thermo scientific glass plates and sealed with coverslips. Samples were kept in the dark before confocal imaging by fluorescent microscopy LSM710 confocal microscope (Zeiss). The electro-spun PCL fibers were also analyzed by SEM with a low-vacuum secondary electron detector (Nova NanoSEM 600™; FEI Zürich, Switzerland). For this purpose, samples were washed twice in PBS and fixed in 2.5% (v/v) glutaraldehyde, 0.1 M sodium cacodylate, pH 7.4 at 4˚C overnight. To overcome reduced electrical conductivity, the samples were dehydrated in serial-grade ethanol (50-99%), followed by solvent removal in the vacuum chamber. The release of Glacial Blue beads incorporated within electro-spun PCL fibers to the culture medium was investigated by using FACSAria III (BD Biosciences, Franklin Lakes, NJ) with a 375-nm laser to excite the beads and emission collected in a 450/50 nm filter. Samples (500 μl) of the medium were collected from the cultures in 24-well plates (Sigma) through Day 1-7. Each sample was passed through the instrument for 120 s. Collected raw data were analyzed by using FlowJo version 9.7.6 (FlowJo LLC, Ashland, OR).

### Quantification of complement sC5b-9 by time-resolved immunofluorometric assay

The potential of PCL-induced complement activation was investigated by measuring soluble membrane attack complex (sMAC). PCL plates were prepared by melting PCL particles at 100°C on a borosilicate glass plate, then cooling to below -30°C to thermally induce solidification. The PCL samples were sterilized for 3 h in 70% ethanol. PCL samples of 50-kDa PCL particles (~15 mg) and PCL plates (~15 mg) were incubated in 150 µl human serum at 37°C for 5, 15, and 30 min. After incubation, a total volume of 25 µl for each sample was used to determine the sC5b-9 concentration using time-resolved immunofluorometric assay (TRIFMA) as previously described (79). Briefly, microtitre wells were coated in 1 µg/ml mouse anti-human sC5b-9 (A711, Quidel, San Diego, CA) and blocked in PBS with BSA. After washing in PBS with Tween 20, PCL/serum samples were diluted four-fold in PBS/Tw containing 10 mM EDTA and incubated overnight at 4°C. A standard was made from normal human serum and activated by incubation with human IgG-Sepharose for 1 h at 37°C. The concentration of sC5b-9 was quantified by comparison with recombinant sC5b-9 (#A239, Quidel). Bound sC5b-9 was detected with 0.05 µg/ml biotinylated anti-human C6 antibody (#A219, Quidel). The wells were subsequently incubated with Eu^3+^-labeled streptavidin (1244-360, Perkin Elmer, MA) in PBS with EDTA. Bound Eu was detected by adding an enhancement solution (Perkin Elmer) and reading the time-resolved fluorescence signal on a DELFIA fluorometer (Victor3^®^, Perkin Elmer).

### Quantification of sCD18 by time-resolved immunofluorometry

As previously described, the concentration of sCD18 in culture supernatants was quantified with TRIFMA (66). Briefly, wells were coated with antibodies to CD18 (KIM127 or KIM185) or isotype murine IgG1 and blocked in TBS with human albumin. After washes, samples of 100 μl heparinized plasma diluted 1:10 and 1:5 or supernatants diluted 1:2 in TBS/Tween with 1 mM CaCl_2_, 1 mM MgCl_2_, and 100 μg/ml aggregated human Ig were added to the wells. The plates were incubated overnight at 4°C. After incubation of the diluted samples, the wells were washed and subsequently incubated with 100 μl biotinylated KIM127 antibody. After washing the wells, Eu^3+^-conjugated streptavidin was applied, and the signals were read by time-resolved fluorometry. Signals from *in vitro* samples were compared against a standard curve made from titrations of plasma (≡1 U/ml) from healthy donor controls.

### Confocal microscopy of CD18 adsorption

Cells for CD18 immunocytochemistry were grown in a microenvironment consisting of PCL fibers on cover glasses for seven days and under monocyte-to-monocyte fusion conditions as described above. Confluent cell layers were washed twice in PBS and fixed in 3.7% (v/v) paraformaldehyde for 10 min at RT. Antigen blocking was performed using PBS with 0.3% (v/v) Triton-X100 and 1% (w/v) BSA solution. Mouse and bovine IgG (100 µg/ml) were added and dissolved in PBS for 30 min at RT. The primary murine antibodies to human CD18 were diluted in PBS to a final concentration of 5 µg/ml. Negative controls comprised mouse isotype IgG1 at (5 µg/ml). The secondary antibody was used for the second negative control (goat-anti-mouse at 5 µg/ml). These steps were followed by incubation in the dark for 24 h at 4°C. The secondary antibody was then added (goat-anti-mouse) and incubated for 1 h at RT. Phalloidin-red, diluted at 1:500, was then added and incubated for 5 min at RT. In between the steps, samples were washed 3 times for 5 min in PBS to remove unbound antibodies. The last washing step was divided into two parts, first using PBS twice and then distilled water for rinsing. Finally, samples were mounted on glass slides using a fluorescence medium (Dako).

### DMA of PCL after cellular exposure

The major challenge is gaining controllable morphology, area, and topography precision by electrospinning, in addition to the electro-spun PCL fiber’s potential anisotropic behavior and weak nature as a load-bearing structure. For this reason, compact test specimens for DMA were constructed using molding, as described above. The fabricated test specimens were then compared individually to determine if they had similar properties regarding Young’s modulus, tensile stress, yield stress, and visual appearance. The dynamic vibration technique was applied at RT with the Bose ElectroForce™ 3200 Series II instrument (Bose Corporation, Framingham, MA). It was used to measure Tan delta (δ), i.e., the relationship between loss modulus (E’’) and storage modulus (E’). Test specimens were treated for 3 h in 5 M NaOH solution, followed by disinfection with 70% (v/v) ethanol for 24 h and washed twice in PBS. Baseline measurements were performed for all samples (n=3) before monocyte seeding (3.5×10^5^ cells/cm^2^) in 12-wells Corning^®^ Costar^®^ cell culture plates (Sigma) with MEM containing 25 ng/ml M-CSF, 40 ng/ml RANK-L and 5 ng/ml TGF-β3 dissolved in αMEM with 10% (v/v) FCS. As a control, specimens were exposed to a complete medium without cells. Cultures were kept in a humidified atmosphere with 5% CO_2_ at 37°C for periods of 1, 2, or 3 weeks. Low energy radiation-mediated degradation or ultraviolet light exposure was avoided throughout the period. The above-noted dynamic mechanical analysis measured tan δ, and the frequencies applied ranged from 1–200 Hz. The mean value of the amplitude of displacement equaled 0.10 mm.

### Statistics

Statistical analysis was performed using STATA 12 (StataCorp LP, College Station, TX) and GraphPad Prism 5 (GraphPad Software Inc., La Jolla, CA). Data was analyzed as one sample from a normal distribution based on paired or unpaired t-tests for *in vivo* and *in vitro* observations. Further, one-way ANOVA analysis was applied when comparing the means of at least three conditions in a particular experiment, where the observations are independent, and the populations have a comparable standard deviation. The normality assumption was investigated by creating histograms and inverse normal or Quantile-Quantile (QQ) plots for raw data and after logarithmic transformation. A non-parametric Wilcoxon-Mann-Whitney test was used for a non-Gaussian distribution when comparing two populations. A p-value of < 0.05 represents a statistically significant difference between means.

## Data Availability

The original contributions presented in the study are included in the article/**Supplementary Material**. Further inquiries can be directed to the corresponding authors.

## References

[1] Kirmanidou Y Chatzinikolaidou M Michalakis K Tsouknidas A. Clinical translation of polycaprolactone-based tissue engineering scaffolds, fabricated via additive manufacturing: A review of their craniofacial applications. Biomater Adv. (2024) 162:213902. doi: 10.1016/j.bioadv.2024.213902 38823255

[2] Belzberg M Mitchell KA Ben-Shalom N Asemota AO Wolff AY Santiago GF. Cranioplasty outcomes from 500 consecutive neuroplastic surgery patients. J Craniofac Surg. (2022) 33(6):1648–54. doi: 10.1097/SCS.0000000000008546 35245275

[3] Khalid SI Thomson KB Maasarani S Wiegmann AL Smith J Adogwa O. Materials used in cranial reconstruction: A systematic review and meta-analysis. World Neurosurg. (2022) 164:e945–63. doi: 10.1016/j.wneu.2022.05.073 35623608

[4] Woodruff MA Hutmacher DW. The return of a forgotten polymer—Polycaprolactone in the 21st century. Prog Polymer Sci. (2010) 35:1217–56. doi: 10.1016/j.progpolymsci.2010.04.002

[5] Mittal V Akhtar T Matsko N. Mechanical, thermal, rheological and morphological properties of binary and ternary blends of PLA, TPS and PCL. Macromol Mater Eng. (2015) 300:423–35. doi: 10.1002/mame.201400332

[6] Verma M Vishwanath K Eweje F Roxhed N Grant T Castaneda M. A gastric resident drug delivery system for prolonged gram-level dosing of tuberculosis treatment. Sci Transl Med. (2019) 11. doi: 10.1126/scitranslmed.aau6267 PMC7797620 30867322

[7] Rashid M Dudhia J Dakin SG Snelling SJB De Godoy R Mouthuy P-A. Histopathological and immunohistochemical evaluation of cellular response to a woven and electrospun polydioxanone (PDO) and polycaprolactone (PCL) patch for tendon repair. Sci Rep. (2020) 10:4754. doi: 10.1038/s41598-020-61725-5 32179829 PMC7076042

[8] Kamath SM Sridhar K Jaison D Gopinath V Ibrahim BKM Gupta N. Fabrication of tri-layered electrospun polycaprolactone mats with improved sustained drug release profile. Sci Rep. (2020) 10:18179. doi: 10.1038/s41598-020-74885-1 33097770 PMC7584580

[9] Shkarina S Shkarin R Weinhardt V Melnik E Vacun G Kluger PJ. 3D biodegradable scaffolds of polycaprolactone with silicate-containing hydroxyapatite microparticles for bone tissue engineering: high-resolution tomography and *in vitro* study. Sci Rep. (2018) 8:8907. doi: 10.1038/s41598-018-27097-7 29891842 PMC5995873

[10] Black C Kanczler JM de Andrés MC White LJ Savi FM Bas O. Characterisation and evaluation of the regenerative capacity of Stro-4+ enriched bone marrow mesenchymal stromal cells using bovine extracellular matrix hydrogel and a novel biocompatible melt electro-written medical-grade polycaprolactone scaffold. Biomaterials. (2020) 247:119998. doi: 10.1016/j.biomaterials.2020.119998 32251928 PMC7184676

[11] Mondal D Griffith M Venkatraman SS. Polycaprolactone-based biomaterials for tissue engineering and drug delivery: Current scenario and challenges. Inter J Polymer Mater Polymer Biomater. (2016) 65:255–65. doi: 10.1080/00914037.2015.1103241

[12] Schantz JT Lim TC Ning C Teoh SH Tan KC Wang SC. Cranioplasty after trephination using a novel biodegradable burr hole cover: technical case report. Neurosurgery. (2006) 58:ONS–E176. doi: 10.1227/01.NEU.0000193533.54580.3F 16462619

[13] Cho SW Shin BH Heo CY Shim JH. Efficacy study of the new polycaprolactone thread compared with other commercialized threads in a murine model. J Cosmet Dermatol. (2021) 20:2743–9. doi: 10.1111/jocd.13883 PMC8451902 33421303

[14] Malinauskas M Jankauskaite L Aukstikalne L Dabasinskaite L Rimkunas A Mickevicius T. Cartilage regeneration using improved surface electrospun bilayer polycaprolactone scaffolds loaded with transforming growth factor-beta 3 and rabbit muscle-derived stem cells. Front Bioeng Biotechnol. (2022) 10:971294. doi: 10.3389/fbioe.2022.971294 36082160 PMC9445302

[15] Jensen BN Wang Y Le Friec A Nabavi S Dong M Seliktar D. Wireless electromagnetic neural stimulation patch with anisotropic guidance. NPJ Flexible Electron. (2023) 7:34. doi: 10.1038/s41528-023-00270-3

[16] Morales-Gomez JA Garcia-Estrada E Leos-Bortoni JE Delgado-Brito M Flores-Huerta LE de la Cruz-Arriaga AA. Cranioplasty with a low-cost customized polymethylmethacrylate implant using a desktop 3D printer. J Neurosurg. (2018) 130:1721–7. doi: 10.3171/2017.12.JNS172574 29905512

[17] Park H Choi JW Jeong WS. Clinical application of three-dimensional printing of polycaprolactone/beta-tricalcium phosphate implants for cranial reconstruction. J Craniofac Surg. (2022) 33:1394–9. doi: 10.1097/SCS.0000000000008595 PMC9275841 35261367

[18] Le BQ Rai B Hui Lim ZX Tan TC Lin T Lin Lee JJ. A polycaprolactone-beta-tricalcium phosphate-heparan sulphate device for cranioplasty. J Craniomaxillofac Surg. (2019) 47:341–8. doi: 10.1016/j.jcms.2018.11.013 30579746

[19] Koo HT Oh J Heo CY. Cranioplasty using three-dimensional-printed polycaprolactone implant and free latissimus dorsi musculocutaneous flap in a patient with repeated wound problem following titanium cranioplasty. Arch Plast Surg. (2022) 49:740–4. doi: 10.1055/s-0042-1748656 PMC9747279 36523917

[20] He L. Biomaterials for regenerative cranioplasty: current state of clinical application and future challenges. J Funct Biomater. (2024) 15. doi: 10.3390/jfb15040084 PMC11050949 38667541

[21] Hwang K Villavicencio JB Agdamag AMP. Tissue engineering and regenerative medicine cranioplasty using polycaprolactone-tricalcium phosphate: management and treatment outcomes. Neurosurgery Pract. (2021) 2:okab027. doi: 10.1093/neuopn/okab027

[22] Fuchs A Bartolf-Kopp M Bohm H Straub A Kubler AC Linz C. Composite grafts made of polycaprolactone fiber mats and oil-based calcium phosphate cement pastes for the reconstruction of cranial and maxillofacial defects. Clin Invest. (2023) 27:3199–209. doi: 10.1007/s00784-023-04932-4 PMC10264493 36864278

[23] Jungebluth P Alici E Baiguera S Blomberg P Bozoky B Crowley C. Tracheobronchial transplantation with a stem-cell-seeded bioartificial nanocomposite: a proof-of-concept study. Lancet. (2011) 378:1997–2004. doi: 10.1016/S0140-6736(11)61715-7 22119609

[24] Cyranoski D. Surgeon commits misconduct. Nature. (2015) 521:406–7. doi: 10.1038/nature.2015.17605 26017424

[25] Park HS Lee JS Jung H Kim DY Kim SW Sultan MT. An omentum-cultured 3D-printed artificial trachea: *in vivo* bioreactor. Artif Cells Nanomed Biotechnol. (2018) 46:S1131–40. doi: 10.1080/21691401.2018.1533844 30451550

[26] Woodward SC Brewer PS Moatamed F Schindler A Pitt CG. The intracellular degradation of poly(epsilon-caprolactone). J BioMed Mater Res. (1985) 19:437–44. doi: 10.1002/jbm.820190408 4055826

[27] Chambers TJ. Multinucleate giant cells. J Pathol. (1978) 126:125–48. doi: 10.1002/path.1711260302 370353

[28] Udagawa N Takahashi N Akatsu T Tanaka H Sasaki T Nishihara T. Origin of osteoclasts: mature monocytes and macrophages are capable of differentiating into osteoclasts under a suitable microenvironment prepared by bone marrow-derived stromal cells. Proc Natl Acad Sci U S A. (1990) 87:7260–4. doi: 10.1073/pnas.87.18.7260 PMC54723 2169622

[29] Goyert SM Ferrero E Rettig WJ Yenamandra AK Obata F Le Beau MM. The CD14 monocyte differentiation antigen maps to a region encoding growth factors and receptors. Science. (1988) 239:497–500. doi: 10.1126/science.2448876 2448876

[30] Siparsky GL Voorhees KJ Miao F. Hydrolysis of polylactic acid (PLA) and polycaprolactone (PCL) in aqueous acetonitrile solutions: autocatalysis. J Environ Polymer Degrad. (1998) 6:31–41. doi: 10.1023/A:1022826528673

[31] Lee BS Holliday LS Ojikutu B Krits I Gluck SL. Osteoclasts express the B2 isoform of vacuolar H(+)-ATPase intracellularly and on their plasma membranes. Am J Physiol. (1996) 270:C382–8. doi: 10.1152/ajpcell.1996.270.1.C382 8772466

[32] Uribe-Querol E Rosales C. Phagocytosis: our current understanding of a universal biological process. Front Immunol. (2020) 11:1066. doi: 10.3389/fimmu.2020.01066 32582172 PMC7280488

[33] Madel M-B Ibáñez L Wakkach A de Vries TJ Teti A Apparailly F. Immune function and diversity of osteoclasts in normal and pathological conditions. Front Immunol. (2019) 10. doi: 10.3389/fimmu.2019.01408 PMC6594198 31275328

[34] Zoltowska K Sobczak M Oledzka E. Novel zinc-catalytic systems for ring-opening polymerization of epsilon-caprolactone. Molecules. (2015) 20:2816–27. doi: 10.3390/molecules20022816 PMC6272648 25690282

[35] Wilkes RA Aristilde L. Degradation and metabolism of synthetic plastics and associated products by Pseudomonas sp. capabilities challenges. J Appl Microbiol. (2017) 123:582–93. doi: 10.1111/jam.2017.123.issue-3 28419654

[36] Ries WL. Osteogenic periosteum esterase activity: a comparative morphological and cytochemical study of bone cells in *situ* on rat proximal tibiae and in smears. J Histochem Cytochem. (1984) 32:55–62. doi: 10.1177/32.1.6197438 6197438

[37] Vorup-Jensen T Carman CV Shimaoka M Schuck P Svitel J Springer TA. Exposure of acidic residues as a danger signal for recognition of fibrinogen and other macromolecules by integrin alphaXbeta2. Proc Natl Acad Sci U S A. (2005) 102:1614–9. doi: 10.1073/pnas.0409057102 PMC547869 15665082

[38] Vorup-Jensen T Jensen RK. Structural immunology of complement receptors 3 and 4. Front Immunol. (2018) 9:2716. doi: 10.3389/fimmu.2018.02716 30534123 PMC6275225

[39] Juul-Madsen K Qvist P Bendtsen KL Langkilde AE Vestergaard B Howard KA. Size-Selective Phagocytic Clearance of Fibrillar alpha-Synuclein through Conformational Activation of Complement Receptor 4. J Immunol. (2020) 204:1345–61. doi: 10.4049/jimmunol.1900494 31969389

[40] Juul-Madsen K Parbo P Ismail R Ovesen PL Schmidt V Madsen LS. Amyloid-beta aggregates activate peripheral monocytes in mild cognitive impairment. Nat Commun. (2024) 15:1224. doi: 10.1038/s41467-024-45627-y 38336934 PMC10858199

[41] Brevig T Holst B Ademovic Z Rozlosnik N Rohrmann JH Larsen NB. The recognition of adsorbed and denatured proteins of different topographies by beta2 integrins and effects on leukocyte adhesion and activation. Biomaterials. (2005) 26:3039–53. doi: 10.1016/j.biomaterials.2004.09.006 15603799

[42] Mohammad-Beigi H Hayashi Y Zeuthen CM Eskandari H Scavenius C Juul-Madsen K. Mapping and identification of soft corona proteins at nanoparticles and their impact on cellular association. Nat Commun. (2020) 11:4535. doi: 10.1038/s41467-020-18237-7 32913217 PMC7484794

[43] Kopp MRG Grigolato F Zurcher D Das TK Chou D Wuchner K. Surface-induced protein aggregation and particle formation in biologics: current understanding of mechanisms, detection and mitigation strategies. J Pharm Sci. (2023) 112:377–85. doi: 10.1016/j.xphs.2022.10.009 36223809

[44] Bajic G Yatime L Sim RB Vorup-Jensen T Andersen GR. Structural insight on the recognition of surface-bound opsonins by the integrin I domain of complement receptor 3. Proc Natl Acad Sci U S A. (2013) 110:16426–31. doi: 10.1073/pnas.1311261110 PMC3799375 24065820

[45] Vasconcelos DM Goncalves RM Almeida CR Pereira IO Oliveira MI Neves N. Fibrinogen scaffolds with immunomodulatory properties promote *in vivo* bone regeneration. Biomaterials. (2016) 111:163–78. doi: 10.1016/j.biomaterials.2016.10.004 27728815

[46] Takayanagi H. Osteoimmunology: shared mechanisms and crosstalk between the immune and bone systems. Nat Rev Immunol. (2007) 7:292–304. doi: 10.1038/nri2062 17380158

[47] Pacifici R. T cells, osteoblasts, and osteocytes: interacting lineages key for the bone anabolic and catabolic activities of parathyroid hormone. Ann N Y Acad Sci. (2016) 1364:11–24. doi: 10.1111/nyas.2016.1364.issue-1 26662934 PMC4803611

[48] Stapulionis R Oliveira CL Gjelstrup MC Pedersen JS Hokland ME Hoffmann SV. Structural insight into the function of myelin basic protein as a ligand for integrin alpha M beta 2. J Immunol. (2008) 180:3946–56. doi: 10.4049/jimmunol.180.6.3946 18322203

[49] Underhill DM Goodridge HS. Information processing during phagocytosis. Nat Rev Immunol. (2012) 12:492–502. doi: 10.1038/nri3244 22699831 PMC5570470

[50] Bartnikowski M Dargaville TR Ivanovski S Hutmacher DW. Degradation mechanisms of polycaprolactone in the context of chemistry, geometry and environment. Prog Polymer Sci. (2019) 96:1–20. doi: 10.1016/j.progpolymsci.2019.05.004

[51] Wissing TB Bonito V van Haaften EE van Doeselaar M Brugmans M Janssen HM. Macrophage-driven biomaterial degradation depends on scaffold microarchitecture. Front Bioeng Biotechnol. (2019) 7:87. doi: 10.3389/fbioe.2019.00087 31080796 PMC6497794

[52] Ganda S Wong CK Biazik J Raveendran R Zhang L Chen F. Macrophage-targeting and complete lysosomal degradation of self-assembled two-dimensional poly(epsilon-caprolactone) platelet particles. ACS Appl Mater Interfaces. (2022) 14:35333–43. doi: 10.1021/acsami.2c06555 35895018

[53] Khan UA Hashimi SM Bakr MM Forwood MR Morrison NA. Foreign body giant cells and osteoclasts are TRAP positive, have podosome-belts and both require OC-STAMP for cell fusion. J Cell Biochem. (2013) 114:1772–8. doi: 10.1002/jcb.v114.8 23444125

[54] McNally AK Anderson JM. Interleukin-4 induces foreign body giant cells from human monocytes/macrophages. Differential lymphokine regulation of macrophage fusion leads to morphological variants of multinucleated giant cells. Am J Pathol. (1995) 147:1487–99. Available at: https://pmc.ncbi.nlm.nih.gov/articles/PMC1869534/PMC1869534 7485411

[55] Kao WJ McNally AK Hiltner A Anderson JM. Role for interleukin-4 in foreign-body giant cell formation on a poly(etherurethane urea) *in vivo* . J BioMed Mater Res. (1995) 29:1267–75. doi: 10.1002/jbm.820291014 8557729

[56] Rodriguez A Macewan SR Meyerson H Kirk JT Anderson JM. The foreign body reaction in T-cell-deficient mice. J BioMed Mater Res A. (2009) 90:106–13. doi: 10.1002/jbm.a.v90a:1 PMC3864678 18491378

[57] Arron JR Choi Y. Bone versus immune system. Nature. (2000) 408:535–6. doi: 10.1038/35046196 11117729

[58] Miron RJ Zohdi H Fujioka-Kobayashi M Bosshardt DD. Giant cells around bone biomaterials: Osteoclasts or multi-nucleated giant cells? Acta Biomater. (2016) 46:15–28. doi: 10.1016/j.actbio.2016.09.029 27667014

[59] Miron RJ Bosshardt DD. Multinucleated giant cells: good guys or bad guys? Tissue Eng Part B Rev. (2018) 24:53–65. doi: 10.1089/ten.TEB.2017.0242 28825357

[60] Ahmadzadeh K Vanoppen M Rose CD Matthys P Wouters CH. Multinucleated giant cells: current insights in phenotype, biological activities, and mechanism of formation. Front Cell Dev Biol. (2022) 10:873226. doi: 10.3389/fcell.2022.873226 35478968 PMC9035892

[61] ten Harkel B Schoenmaker T Picavet DI Davison NL de Vries TJ Everts V. The foreign body giant cell cannot resorb bone, but dissolves hydroxyapatite like osteoclasts. PLoS One. (2015) 10:e0139564. doi: 10.1371/journal.pone.0139564 26426806 PMC4591016

[62] Ponzetti M Rucci N. Updates on osteoimmunology: what’s new on the cross-talk between bone and immune system. Front Endocrinol. (2019) 10:236. doi: 10.3389/fendo.2019.00236 PMC6482259 31057482

[63] Vorup-Jensen T Peer D. Nanotoxicity and the importance of being earnest. Adv Drug Delivery Rev. (2012) 64:1661–2. doi: 10.1016/j.addr.2012.09.002 23000008

[64] Simberg D Park JH Karmali PP Zhang WM Merkulov S McCrae K. Differential proteomics analysis of the surface heterogeneity of dextran iron oxide nanoparticles and the implications for their *in vivo* clearance. Biomaterials. (2009) 30:3926–33. doi: 10.1016/j.biomaterials.2009.03.056 PMC2792080 19394687

[65] Evans BJ McDowall A Taylor PC Hogg N Haskard DO Landis RC. Shedding of lymphocyte function-associated antigen-1 (LFA-1) in a human inflammatory response. Blood. (2006) 107:3593–9. doi: 10.1182/blood-2005-09-3695 16418329

[66] Gjelstrup LC Boesen T Kragstrup TW Jorgensen A Klein NJ Thiel S. Shedding of large functionally active CD11/CD18 Integrin complexes from leukocyte membranes during synovial inflammation distinguishes three types of arthritis through differential epitope exposure. J Immunol. (2010) 185:4154–68. doi: 10.4049/jimmunol.1000952 20826754

[67] Gomez IG Tang J Wilson CL Yan W Heinecke JW Harlan JM. Metalloproteinase-mediated Shedding of Integrin beta2 promotes macrophage efflux from inflammatory sites. J Biol Chem. (2012) 287:4581–9. doi: 10.1074/jbc.M111.321182 PMC3281606 22170060

[68] Stoy S Sandahl TD Hansen AL Deleuran B Vorup-Jensen T Vilstrup H. Decreased monocyte shedding of the migration inhibitor soluble CD18 in alcoholic hepatitis. Clin Transl Gastroenterol. (2018) 9:160. doi: 10.1038/s41424-018-0022-7 29904132 PMC6002386

[69] Ferapontov A Mellemkjaer A McGettrick HM Vorup-Jensen T Kragstrup TW Juul-Madsen K. Large soluble CD18 complexes with exclusive ICAM-1-binding properties are shed during immune cell migration in inflammation. J Transl Autoimmun. (2025) 10:100266. doi: 10.1016/j.jtauto.2025.100266 39867459 PMC11759538

[70] Kragstrup TW Jalilian B Hvid M Kjaergaard A Ostgard R Schiottz-Christensen B. Decreased plasma levels of soluble CD18 link leukocyte infiltration with disease activity in spondyloarthritis. Arthritis Res Ther. (2014) 16:R42. doi: 10.1186/ar4471 24490631 PMC3978678

[71] Feng S Yue Y Chen J Zhou J Li Y Zhang Q. Biodegradation mechanism of polycaprolactone by a novel esterase MGS0156: a QM/MM approach. Environ Sci Process Impacts. (2020) 22:2332–44. doi: 10.1039/D0EM00340A 33146659

[72] Vorup-Jensen T Boesen T. Protein ultrastructure and the nanoscience of complement activation. Adv Drug Delivery Rev. (2011) 63:1008–19. doi: 10.1016/j.addr.2011.05.023 21699938

[73] McHugh KP Hodivala-Dilke K Zheng MH Namba N Lam J Novack D. Mice lacking beta3 integrins are osteosclerotic because of dysfunctional osteoclasts. J Clin Invest. (2000) 105:433–40. doi: 10.1172/JCI8905 PMC289172 10683372

[74] Heneka MT Carson MJ El Khoury J Landreth GE Brosseron F Feinstein DL. Neuroinflammation in alzheimer’s disease. Lancet Neurol. (2015) 14:388–405. doi: 10.1016/S1474-4422(15)70016-5 25792098 PMC5909703

[75] Jensen J Rolfing JH Le DQ Kristiansen AA Nygaard JV Hokland LB. Surface-modified functionalized polycaprolactone scaffolds for bone repair: *in vitro* and *in vivo* experiments. J BioMed Mater Res A. (2014) 102:2993–3003. doi: 10.1002/jbm.a.v102.9 24123983

[76] Frost HM. Bone histomorphometry: choice of marking agent and labelling schedule. In: Recker R, editor. Bone Histomorphometry. Techniques and Interpretation. CRC Press, Boca Raton (1983). p. 37–51.

[77] Susa M Luong-Nguyen NH Cappellen D Zamurovic N Gamse R. Human primary osteoclasts: *in vitro* generation and applications as pharmacological and clinical assay. J Transl Med. (2004) 2:6. doi: 10.1186/1479-5876-2-6 15025786 PMC394349

[78] White JM Goodis HE Marshall SJ Marshall GW. Sterilization of teeth by gamma radiation. J Dent Res. (1994) 73:1560–7. doi: 10.1177/00220345940730091201 7929992

[79] Haahr-Pedersen S Bjerre M Flyvbjerg A Mogelvang R Dominquez H Hansen TK. Level of complement activity predicts cardiac dysfunction after acute myocardial infarction treated with primary percutaneous coronary intervention. J Invasive Cardiol. (2009) 21:13–9. 19126922

