# Supplementary material for: Osteoclast-like multinucleated giant cells reinforce polycaprolactone grafts
Source: Front Immunol. 2025 May 21;16:1572238. doi: 10.3389/fimmu.2025.1572238 (PMC12133856; doi:10.3389/fimmu.2025.1572238)
Supplement: Supplementary file 1 [file DataSheet1.docx]

Supplementary Materials for

**Osteoclast-like multinucleated giant cells reinforce polycaprolactone grafts**

Halldór Bjarki Einarsson, Anders Frisk Mortensen, Morten Schallburg Nielsen, Menglin Chen, Søren Roesgaard Nielsen, David Christian Evar Kraft, Jonas Jensen, Mette Bjerre,

Morten Nørregaard Andersen, Jens Vinge Nygaard, Cody Eric Bünger,

Thomas Vorup-Jensen

*Correspondence to: [h.einarsson@rn.dk](mailto:h.einarsson@rn.dk) or [vorup-jensen@biomed.au.dk](mailto:vorup-jensen@biomed.au.dk)

**This PDF file includes:**

Suppl. Figs. 1-6

Suppl. Video Legends 1 & 2

Suppl. Materials & Methods

Suppl. References

Supplementary Figures

**Supplementary Fig. 1**


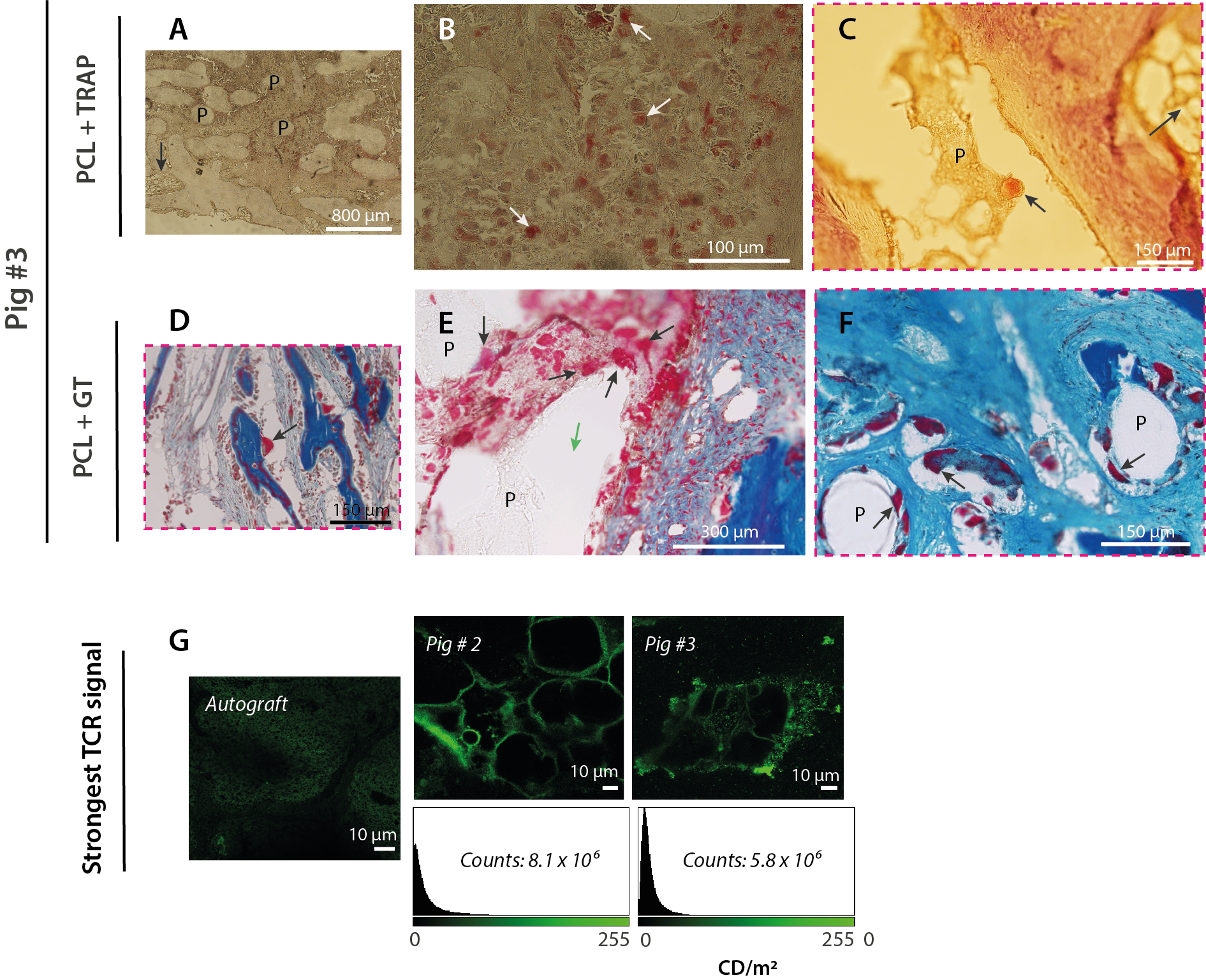


**Histological analyses of PCL in bone microenvironment.** **A-F**, Micrographs shown made by light microscopy imaging of PCL in a convex skull bone (Pig #3) at magnification ×4 versus ×20. TRAP-stained specimens after 8 weeks of PCL implantation showing abundant OC-like infiltration (**A** and **B** [white arrows]) in close proximity to PCL (indicated with P). The manifestation of PCL columns within all specimens is scattered between the trabecular bone. The bone marrow tissue itself is indicated for comparison (**A,** black arrow left and **C,** black arrow right). Infiltrative TRAP^+^ cell (**C**, black arrow left) detected by using oil immersion lens at magnification × 100 in a structure resembling PCL fiber or PCL debris (P) under trabecular bone structure. In comparison the specimens were even found richer in GT+ cells at the side of PCL implantation. These MNGC (**D**-**F**, black arrows) can be seen both at the surface of trabecular bone as characterized for OC (**d**, black arrow) or, when near or attached to PCL (green arrow and P) as red clusters **(E,F)**, *i.e.*, corresponding to an inflammation-zone. Consequently, the histology indicates a cellular level a step further towards differentiated OC-like cells due to PCL exposure. This understanding is also supported by the repeated and strong TCR signal at the side of inflammation zone compared to autograft in Pigs #2 & #3 repeating the results from Pig #1 **(G)**.

**Supplementary Fig. 2**


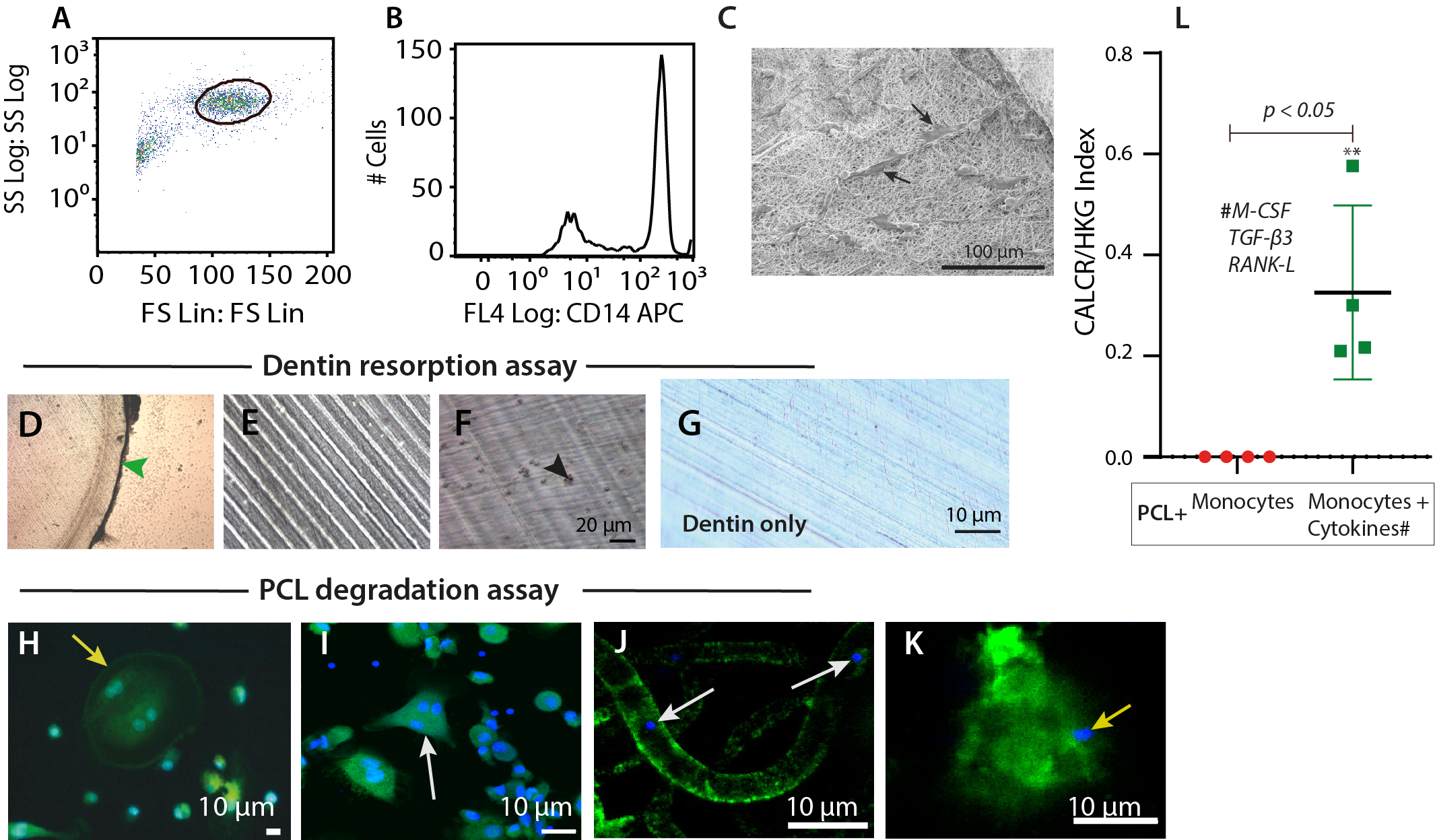


**Characterization of cellular and surface sources. A,B,** A purity of >95% for precursors was gained by the negative selection method (**A**). Two main subpopulations of monocytes were found (**B**, CD14^dim^ and CD14^+^). **c,** SEM imaging gained at HV (5.00 kV) and magnification ×1000 showing MNGC coiling around a PCL fiber (black arrows) in a T cell co-culture. **D-G,** Micrographs of dentin. Light microscopy imaging of a 350-µm thick dentin slice with a cell density at 3.5×10^5^ cells/cm^2^ after 168 h of culture prior to Coomassie staining. The axial image at the magnification ×4 shows the dentin slice is bordered by an enamel and a periodontal ligament (**D**, green arrow). Beneath the enamel, and at increased magnification ×40, longitudinal artefacts made by the microtom are shown as white grooves (**E**). For the same magnification (at ×40), a multinucleated cell is present and attached to dentin (black arrow) confirming the non-toxic condition before the Coomassie and cell trypsinization after 168 hours of cell culturing (**F**). This control testing indicates moreover that the dentin sterilization method by using gamma radiation was sufficient. Negative control dentin at the magnification × 40 after Coomassie staining (**G**). **H,I** After cell culturing with the addition of M-CSF, TGF-β3 and RANK-L for 168 h, a cell viability assay with Cell Tracker™ Green was performed, also counterstained with Hoechst **(H)** or with DAPI **(I)**. The multinucleated giant cells (yellow, **H**, and white arrows, **I**) are shown at magnification ×20. This primary viability assay showed no cell confluency. However, the cell viability tests in our following experiments, revealed that near-confluency can be reached in these experimental setups (Supplementary Fig. 5D,E). **J** ,Microscopic imaging after 168 h showing CD18^+^ PCL fiber with fluorescent beads (white arrows) indicating that the signal gained is from adhesion molecules on the PCL substrate after cell attachment and/or due to CD18-integrin shedding. **K,** CD18^+^CD14^+^ cell after 168 h with engulfed fluoresecent bead (yellow arrow) from a PCL fiber captured by 2D confocal imaging at magnification ×20. This finding was further supported by our 3D confocal imaging. **L,** PCL-associated, cytokine-generated MNGC were confirmed calcitonin receptor (CALCR) positive by RT-PCR from one donor with a CALCR/housekeeping keeping genes index of ~0.33 ± 0.09 (mean±SEM) after 168 h compared to a mean of ~1.17×10^-5^ for cells with no cytokine addition (p = 0.0091, n=4 in a two tailed unpaired *t*-test and after normalization against the best keeper index) as detailed in the Suppl. Methods.

**Supplementary Fig. 3**

**
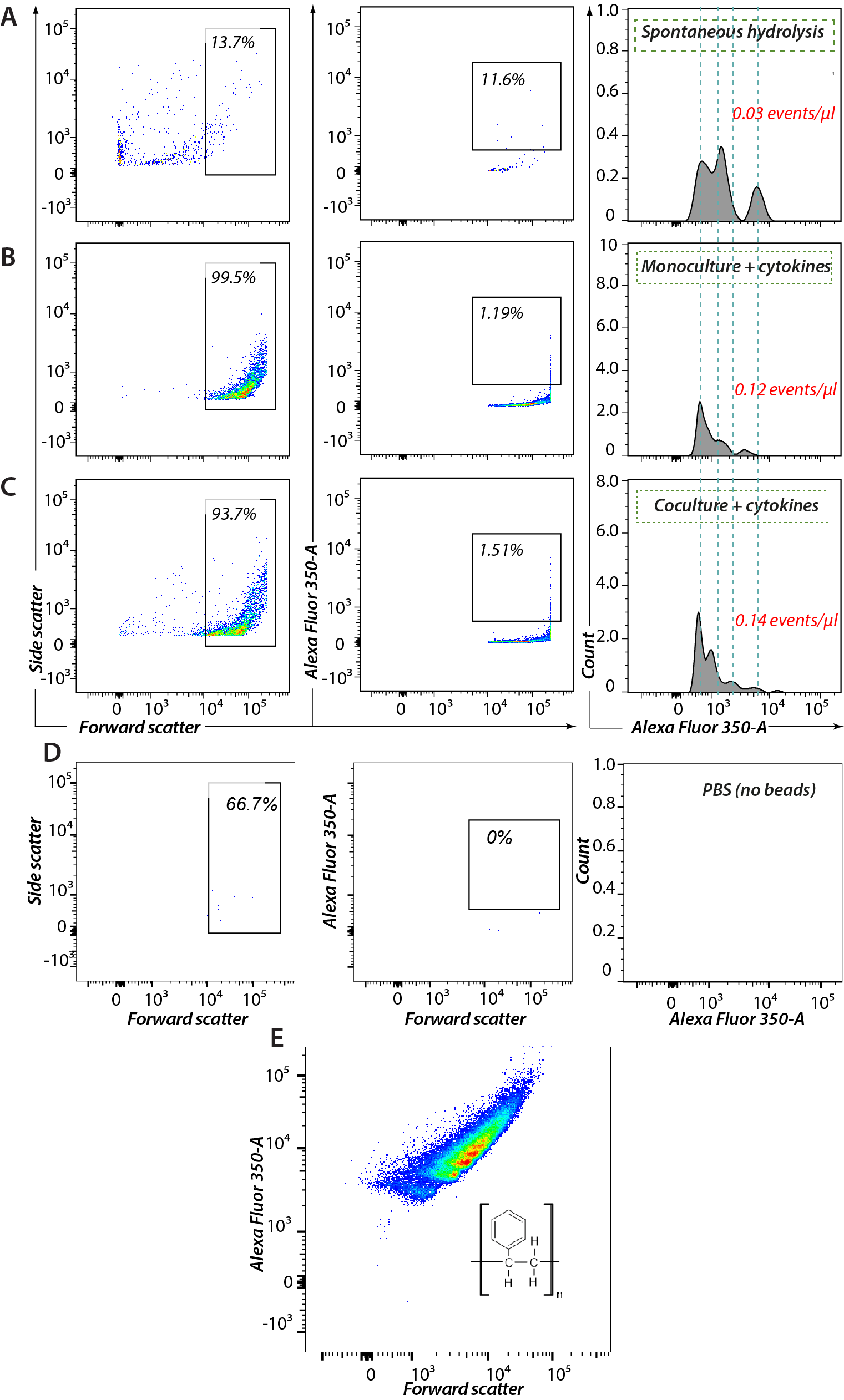
**

***In vitro* release of fluorescent beads from PCL fibers (Donor 2).** **A-C,** Flow cytometric analysis of fragment release from electro-spun PCL fibers by spontaneous hydrolysis, monocytes incubated with cytokines, or co-culture of monocytes and T lymphocytes with cytokines. The contents of the first gate were analyzed in a plot of fluorescence intensity versus forward scatter (size) for the number of fluorescent fiber fragments. A gate was set to enumerate large fragments with robust fluorescence, further plotted as a fluorescence intensity distribution. Hatched grey lines indicated shared features of the fluorescence intensity profile between the experiments. The number of events per volume was calculated from the total events in the gate divided by the sample volume of 500 µl. **D,** As a control, the fluorescence events were investigated for phosphate buffer saline (PBS) alone. **E,** Fluorescence and forward scatter distribution for naked 1-μm GB beads.

**Supplementary Fig. 4**

**
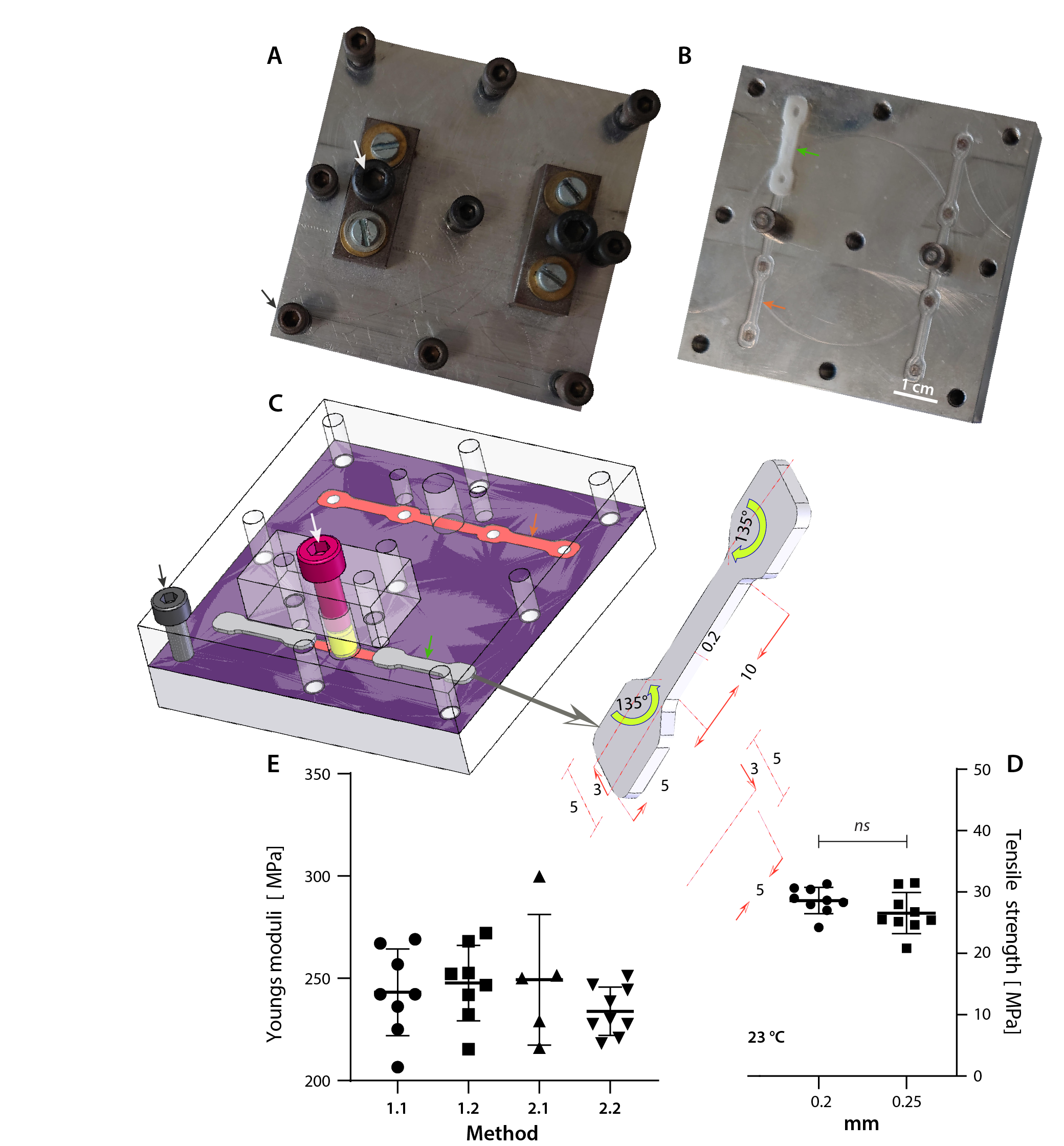
**

**PCL test specimen production for the Dynamic Mechanical Analysis.** **A-C** Mold parts used to prepare the PCL test specimens by injection molding, showing the PCL injection piston (**A**,**C,** white arrows), the lock keyhole between the two mold parts (**A**,**C** black arrows), the PCL cast (**B-C**, orange arrows) and the created PCL specimen (**B-C**, green arrows) with units in mm and the schematically explained geometry (gray arrow). **D-E** PCL test results from static tensile tests revealing the strength and Young’s moduli (stiffness) of the specimens (**E**) between the two different tested PCL thicknesses by the methods used (1.1 to 2.2). The tensile numbers reflect the highest tensile force a specimen can withstand before it breaks (**D**). The results ensured that there was no statistical difference between specimens having two different thicknesses of 0.20 mm and 0.25 mm, receptively, at room temperature. The data was normally distributed (Shapiro-Wilk test), and no significant difference was found between the groups for both variables shown. Tensile strength difference was tested with *t*-test. Difference in Young’s moduli was tested by using one-way ANOVA. For both graphs the SD is shown. The tensile strength values around 25 MPa and the values of Young’s moduli around 240 and 250 MPa are comparable to previous studies on PCL [1]

**Supplementary Fig. 5**

**
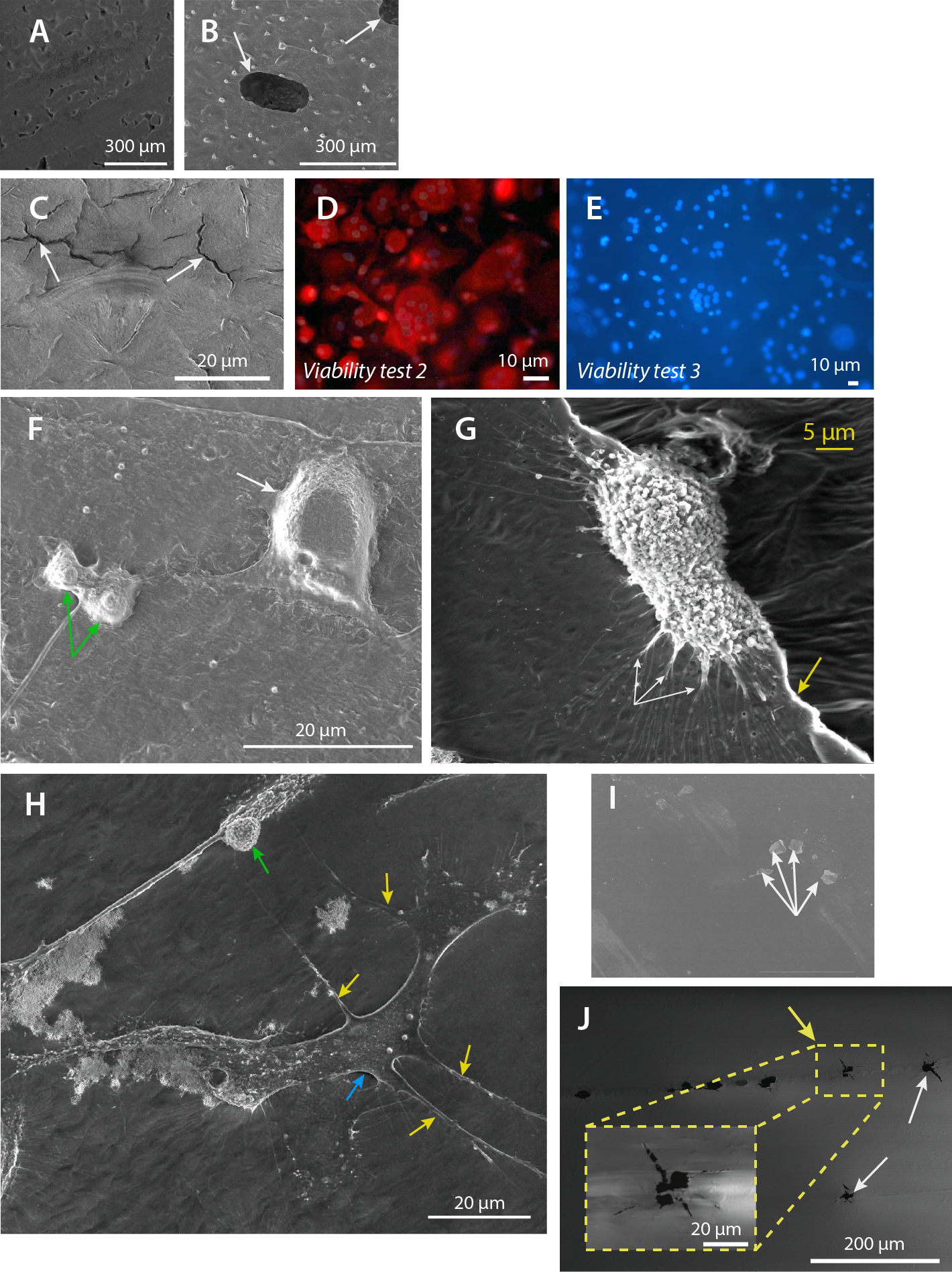
**

**Image evaluation of PCL test specimens after casting or bioplotting and cell exposure**. **A,** SEM at high voltage (4 kV) of a negative PCL control after casting at magnification ×430. The PCL test specimens is characterized by an uneven surface, microcavities, and craters, in addition to surface cracks. **B,** SEM imaging (magnification ×452) at low vacuum mode (3 kV) of bioplotted PCL test specimen, with a homogeneous cell distrubution after co-culturing at 168 h. White arrows indicate large craters. **C** SEM image of PCL (magnification ×5000, 4 kV) test specimen gained from a mold cast with micro-cracks caused by the fabrication method (white arrows). **D,E** In comparison to the primary viability assay (Supplementary Fig. 2h,i), our second and third viability assays confirmed the non-toxic effect of PCL as our experiments (n = 3) revealed cell near-confluency and fusion of CD14^+^ precursor cells, followed by multinucleation after 168 h culture. DAPI staining with Phalloidin-red **(D)** or without **(E)** is shown for comparison at magnification ×40 by using epifluorescent microscope. **F,** SEM imaging (magnification ×3000, low vacuum at 3 kV) of PCL co-culture at 168 h. Lymphocyte-like cells are shown to the left (greenarrows) and monocyte-like cell to the right (white arrow). **G** A similar precursor cell is shown attached to the PCL crater border (yellow arrow) with pseudopodium projections (white arrows) at (magnification ×8247, low vacuum at 5 kV for bioplotted PCL). **H,** For the same specimen, a monocyte derived giant cell is shown in the center and a lymphocyte-like cell in the periphery (green arrow). The image shows pseudopodium projections (yellow arrows) as seen for the precursors. This image (magnification × 2655, low vacuum at 5 kV) also reveals lacunae-like formation (blue arrow) at low vacuum mode (5 kV) after co-culture at 168 h. **I,** SEM image (magnification × 500 at 5 kV) showing a negative control of a bioplotted PCL test specimen with debris (white arrows). **J,** After using the same test specimen fabrication method as in Panel **B, F-I** with the co-culture setup and generation of MNGC for 168 h, a new SEM imaging was performed for comparison. By using the same magnification and high voltage, remaining PCL microcracks are visualized (white arrows). A single PCL crack (raised yellow box) is shown at a higher magnification (×4000). This finding may be a result of either a mechanical induced rupture of micro PCL laminas, hydrolysis, or MNGC indenting of the PCL material.

**Supplementary Fig. 6**

**
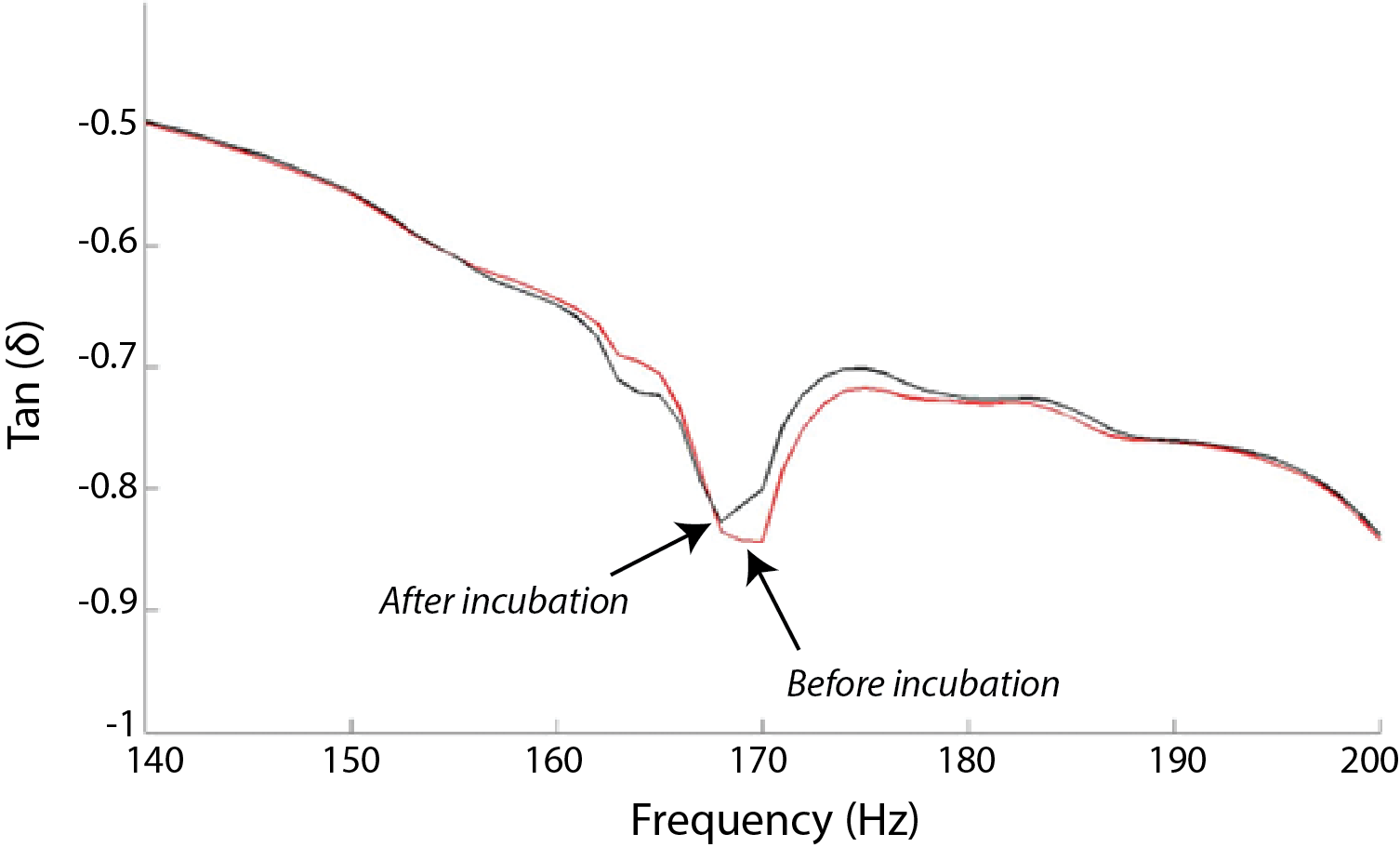
**

**Spontaneous change in PCL specimen during incubations.** Results from DMA of 5 PCL test specimens exposed for 19 days to culturing conditions without cells, comparing the initial specimen properties against the same specimen incubated in the experiment.

**Supplementary Video Legends**

**Video 1**

Confocal imaging of a MNGC plasmalemma intertwined between electro-spun PCL-fibers carrying shed CD18 integrin (green) with embedded fluorescent beads, in blue, multiple cell nuclei, stained red.

**Video 2**

Confocal imaging of a MNGC carrying CD18 integrin. The movie clip shows the intracellular contents by partial removal of the plasmalemma (green). Phagocytosed fluorescent beads below the plasmalemma are indicated in blue. Extracellular beads are indicated in white. The multiple cell nuclei, stained red.

**Supplementary Methods**

*Real-time PCR for the calcitonin receptor*

PBMCs were grown in 48-bottom Corning® Costar® cell culture plates (Sigma) without cover slides. After seven days of culturing, the cells were washed twice with PBS and lysed in a lysis buffer for total RNA extraction (#L8285; Sigma) with 1% β mercaptoethanol (#M3148; Sigma). Cell lysates were kept at -80˚C until further use. RNA was thawed and kept on ice, and the concentration of RNA was measured using a NanoDrop 2000 Spectrophotometer (Thermo Scientific) and adjusted to 50 ng/ml. PCR was performed according to the manufacturer's instructions. The PCR reactions were normalized against the best keeper (BK) index for the household genes RPII (assay ID: Hs00172187_m1), UBC (assay ID: Hs01871556_s1), and HPRT1 6 (assay ID: Hs02800695_m1). Appropriate cDNA dilutions were used subsequently for the RT-7 PCR reactions using specific primers for calcitonin receptor (assay ID: Hs01016882_m1), all from Applied Biosystems, Life Technologies (Thermo Fisher Scientific, USA). RNA was added in 10 duplicates. The qPCR protocol was set up on a Stratagene Mx3005P (Agilent Technologies, 11 USA) with the thermal profile Pre-melt: 48°C for 15 min and 95°C for 10 min. Amplification ×40 at 95°C for 15 s and 60°C for 1 min.

**Suppl. References**

[1] M. Bartnikowski, T.R. Dargaville, S. Ivanovski, D.W. Hutmacher, Degradation mechanisms of polycaprolactone in the context of chemistry, geometry and environment, Progress Polymer Sci 96 (2019) 1-20.
